# Age-associated gut microbiome succession, colonization resistance, and relative resistome patterns in an antibiotic-restricted infant cohort

**DOI:** 10.3389/fmicb.2026.1862116

**Published:** 2026-07-08

**Authors:** Manting Chen, Shutong Zhang, Meiying Lu, Dongyan Zhu, Meiqun Xiao, Ying Liao, Yao Li, Tao Zhou, Mengying Wang, Qiying Song

**Affiliations:** 1 Department of Child Healthcare, Shenzhen Baoan Women's and Children's Hospital, Shenzhen, China; 2 Centre for Big Data Research in Health, Faculty of Medicine and Health, UNSW Sydney, Kensington, NSW, Australia; 3 Medical Research Institute, Shenzhen Baoan Women's and Children's Hospital, Shenzhen, China; 4 Department of Biobank, Shenzhen Baoan Women's and Children's Hospital, Shenzhen, China; 5 Department of Obstetrics, Shenzhen Baoan Women's and Children's Hospital, Shenzhen, China; 6 Department of Epidemiology and Biostatistics, School of Public Health (Shenzhen), Sun Yat-sen University, Shenzhen, China; 7 Department of Nutrition and Food Hygiene, School of Public Health, Peking University, Beijing, China; 8 Key Laboratory of Epidemiology of Major Diseases (Peking University), Ministry of Education, Beijing, China; 9 Health Science Center, Shenzhen University, Shenzhen, China

**Keywords:** antimicrobial resistance, colonization resistance, gut microbiota, microbiome maturation, shotgun metagenomics

## Abstract

**Background:**

Early infancy is critical for gut microbiome assembly and the establishment of colonization resistance against pathobionts. Whether age-associated microbiome maturation is accompanied by changes in colonization-resistance proxies and relative antimicrobial resistance gene profiles under low infant antibiotic exposure remains unclear.

**Methods:**

We analyzed shotgun metagenomes from 82 fecal samples collected from 54 healthy infants (54 at 1 month and 28 at 6 months). Taxonomic and functional profiles were generated using MetaPhlAn 4 and HUMAnN3, and AMR genes were annotated using RGI/CARD. Age-associated taxa were screened by LEfSe and tested using MaAsLin2 with adjustment for key perinatal covariates.

**Results:**

Age group was associated with modest but statistically significant differences in community structure (Bray–Curtis PERMANOVA *R*^2^ = 0.03, *p* = 0.005) and higher species richness at 6 months (*p* < 0.001), with no statistically significant difference in Shannon or Simpson indices. In adjusted models, skin-associated pioneer taxa, including *Staphylococcus epidermidis*, were lower at 6 months, whereas several anaerobic or oral-associated taxa were higher, including *Flavonifractor plautii*. *Enterobacteriaceae* relative abundance was lower at 6 months than at 1 month (median 16.64 vs. 1.86%, *p* < 0.001), and *Bifidobacterium*–*Enterobacteriaceae* antagonism indices were higher. However, *Escherichia coli* and *Klebsiella* spp. did not show significant genus-level reductions. Copies per million (CPM)-normalized β-lactamase (*bla*) relative abundance showed no statistically significant timepoint difference and was positively correlated with selected *Bifidobacterium* species.

**Conclusions:**

In this infant antibiotic-restricted cohort, microbiome profiles at 6 months were associated with lower relative abundance of potential pathobionts and higher colonization-resistance proxy indices. CPM-normalized *bla* relative abundance showed no statistically significant timepoint difference. These observational findings do not establish the genomic host or mobility of *bla* genes. Quantitative and host-resolved studies are needed to distinguish compositional shifts from absolute resistome trajectories.

## 1 Introduction

The human gut microbiota constitutes a complex ecosystem that orchestrates host physiology, immune maturation, and metabolic programming (Rezai et al., 2025; Borrego-Ruiz and Borrego, 2025). Community assembly commences during early infancy, a critical window within the “first 1,000 days" of life. Microbial colonization during this phase has lasting consequences for health and disease susceptibility (Bautista and López-Cortés, 2025; Fahur Bottino et al., 2025).

The infant gut microbiota evolves from a stochastic, low-diversity pioneer community into a stable, resilient ecosystem (Shao et al., 2024; Milani et al., 2017). Although perinatal factors such as delivery mode and feeding practices shape this trajectory (Selma-Royo et al., 2024; Dubois et al., 2024; Zhou et al., 2023), a central functional outcome of microbiome maturation is colonization resistance—the capacity to suppress pathogen and pathobiont overgrowth (Caballero-Flores et al., 2023). A defining feature of this maturation is the compositional shift from facultative to obligate anaerobes (Adlerberth and Wold, 2009; Bäckhed et al., 2015). Initially, the gut is colonized by oxygen-tolerant pathobionts, notably *Enterobacteriaceae* and *Staphylococcaceae* (Adlerberth and Wold, 2009; Shao et al., 2019). As oxygen levels decline, beneficial *Bifidobacterium* species proliferate, fueled by their unique ability to metabolize human milk oligosaccharides (HMOs) (Laursen and Roager, 2023; Ojima et al., 2022). These keystone taxa promote short-chain fatty acid (SCFA) production and suppress *Enterobacteriaceae* expansion, thereby driving the emergence of colonization resistance (Shao et al., 2024; Yang et al., 2024).

Yet several questions remain only partially resolved. First, early-life antibiotic exposure can reshape both microbiome assembly and the infant gut resistome, and the degree of exposure varies widely across clinical settings, delivery practices, and postnatal care pathways (McDonnell et al., 2021; Reyman et al., 2022). Much of the available evidence therefore derives from cohorts with substantial perinatal or postnatal antibiotic exposure, and how age-associated ecological and resistome patterns unfold under minimal direct antibiotic selection remains less well characterized. Second, perinatal factors such as feeding type and delivery mode independently influence microbiome composition (Selma-Royo et al., 2024; Zhou et al., 2023), yet age-associated taxa have not always been evaluated within a covariate-adjusted framework, raising the possibility that dietary or obstetric variation is misattributed to developmental change. Third, whether age-associated ecological maturation is accompanied by a reduction in antibiotic resistance gene (ARG) reservoirs, in the absence of antibiotic intervention, remains insufficiently characterized (Li et al., 2023; Chatzigiannidou et al., 2025).

To address these gaps, we performed shotgun metagenomic sequencing on fecal samples from healthy infants with no documented systemic antibiotic or probiotic exposure before the 6-month visit. This low-exposure profile is a feature of the cohort's eligibility criteria rather than an assumption about regional antibiotic-use patterns. Employing multivariable modeling to adjust for key perinatal and maternal covariates, complemented by paired longitudinal analyses, we aimed to (1) characterize age-associated microbiome differences between 1 and 6 months; (2) quantify colonization-resistance proxy signals using taxon–taxon antagonism metrics; and (3) evaluate whether AMR-related features, particularly copies per million (CPM)-normalized β-lactamase (*bla*) relative abundance, changed in parallel with pathobiont reduction.

## 2 Materials and methods

### 2.1 Study design and participants

This prospective cohort study was conducted at the Shenzhen Baoan Women's and Children's Hospital between November 2020 and October 2021. The study protocol adhered to the ethical guidelines of the 1975 Declaration of Helsinki and was approved by the Ethical Committee of Shenzhen Baoan Women's and Children's Hospital (Approval Number: LLSC 2020-09-02-KS). Written informed consent was obtained from the parents or legal guardians of all participating infants.

We recruited a prospective cohort of 54 healthy mother-infant dyads, enrolling mothers aged 20–45 years with uncomplicated, singleton, term pregnancies (37– <42 weeks). To minimize the confounding effects of medical interventions, we enforced strict exclusion criteria targeting antibiotic exposure and comorbidities. Mothers were excluded if they had pre-existing metabolic or autoimmune disorders or documented antibiotic use during the third trimester. Infants were excluded if they presented with major congenital anomalies, prolonged neonatal intensive care unit (NICU) admission (>48 h), or any documented systemic antibiotic or probiotic exposure before the 6-month visit.

### 2.2 Clinical data collection

Clinical metadata were extracted from electronic medical records and cross-checked through structured maternal interviews. Maternal covariates included demographic and anthropometric profiles, specifically age, pre-pregnancy body mass index (BMI), and gestational weight gain. We also recorded obstetric parameters, including parity, gestational age, and delivery mode. Pregnancy-associated morbidities were rigorously documented, including gestational diabetes mellitus (GDM)—diagnosed via a standard 75-g oral glucose tolerance test (International Association of Diabetes and Pregnancy Study Groups Consensus Panel, 2010) and maternal Group B *Streptococcus* colonization. Neonatal variables, including infant sex and birth weight, were recorded at delivery.

To account for postnatal dietary exposures, infant feeding practices were assessed concurrently with fecal sampling and classified into three categories: exclusive breastfeeding, exclusive formula feeding, and mixed feeding. Mixed feeding was defined as receipt of both breast milk and formula during the sampling period; quantitative information on the proportion of breast milk vs. formula was not collected, and direct breastfeeding was not distinguished from expressed breast milk feeding. Infant exposure to systemic antibiotics or probiotics before the 6-month visit was cross-verified using electronic medical records and structured caregiver interviews. Maternal antibiotic use during the third trimester was recorded from clinical records, whereas maternal antibiotic use during the first 6 months after delivery was not systematically documented.

### 2.3 Sample collection and DNA extraction

Fecal samples were collected at 1 month (*n* = 54) and 6 months of age (*n* = 28), totaling 82 samples. Following collection, samples were immediately placed on ice, transported to the laboratory within 24 h, and archived at −80°C to prevent degradation. Genomic DNA was extracted from approximately 200 mg of fecal material using the QIAamp PowerFecal Pro DNA Kit (Qiagen, Hilden, Germany) following the manufacturer's instructions (Kurnosov et al., 2025). DNA concentration was quantified using Qubit 4.0 fluorometer (Thermo Fisher Scientific, USA), and integrity was verified via agarose gel electrophoresis. Sequencing libraries were constructed using a standard Illumina paired-end library construction protocol. Prior to sequencing, library quality and fragment size distribution were validated. Metagenomic sequencing was conducted on the Illumina NovaSeq 6000 platform (Illumina, San Diego, CA, USA). Sequencing depth and quality metrics were reviewed for all 82 metagenomic samples. Raw read counts ranged from 19.65 to 29.32 million reads per sample, with a mean of 22.49 million reads and a median of 22.10 million reads. Raw sequencing output ranged from 5.93 to 8.85 Gb per sample, with a mean of 6.79 Gb. Sequencing quality was consistently high, with Q30 values ranging from 93.15 to 96.32% (mean 94.53%). No sample was excluded based on sequencing depth after library-level quality control.

### 2.4 Bioinformatic analysis

Raw sequencing reads underwent quality control via fastp (Chen et al., 2018), comprising adapter trimming, low-quality base removal, and filtering of ambiguous reads. To eliminate host contamination, high-quality reads were aligned to the human reference genome using BWA (Li and Durbin, 2009). Host-mapped reads were discarded, retaining non-host reads for downstream analysis. Taxonomic profiling was executed with MetaPhlAn 4 (Blanco-Míguez et al., 2023), based on clade-specific marker genes. Functional potential was characterized via HUMAnN 3 (Beghini et al., 2021). High-quality reads were mapped to the UniRef90 protein database to quantify gene family abundances, which were subsequently stratified into MetaCyc metabolic pathways. ARGs were annotated using the Resistance Gene Identifier (RGI) against the Comprehensive Antibiotic Resistance Database (CARD) (Alcock et al., 2020). CARD/RGI annotations were first restricted to hits classified as “perfect” or “strict." The retained hits were then filtered using thresholds of ≥80% sequence identity and ≥80% alignment coverage (Li et al., 2023).

To enable genome-resolved analysis, high-quality non-host reads were assembled and binned into metagenome-assembled genomes (MAGs) per sample. Reads were assembled with MEGAHIT v1.2.9 (Li et al., 2015), and contigs (≥500 bp) were binned using MetaBAT2 v2.18 (Kang et al., 2019) and MaxBin2 v2.2.7 (Wu et al., 2016). Genome completeness and contamination were estimated with CheckM2 (v1.1.0) (Chklovski et al., 2023), and taxonomy was assigned with GTDB-Tk (v2.7.2) (Chaumeil et al., 2022) using the bacterial 120-marker (bac120) reference set, yielding a catalog of 171 MAGs. To attribute ARGs to specific genomes, RGI was run against CARD on the MAG nucleotide sequences using the same classification and identity/coverage thresholds described above for the read-based annotation; each β-lactamase gene was attributed to the host MAG of the contig on which it was located. Plasmid and proviral (prophage) sequences among the assembled contigs were identified with geNomad (v1.12.0) (Camargo et al., 2024) in end-to-end mode, and each *bla*-carrying contig was cross-referenced against the geNomad plasmid and virus classifications to assess its mobile genetic context.

### 2.5 Statistical analysis

Statistical analysis and data visualization were performed using a hybrid workflow integrating R (v4.3.0) and Python (v3.8) environments on a Linux platform. Ecological diversity was assessed using the R package *vegan* (Oksanen et al., 2022). Alpha diversity indices, including observed richness, Shannon index, and Simpson index, were computed to quantify within-sample diversity. Beta diversity was summarized using Bray–Curtis dissimilarity, visualized by principal coordinates analysis (PCoA), and statistically evaluated with PERMANOVA (999 permutations) (Anderson, 2001).

To formally assess potential attrition bias, baseline maternal and infant characteristics at 1 month of age were compared between the retained cohort (infants with paired 6-month samples; *n* = 28) and the non-retained cohort (infants lost to follow-up; *n* = 26). Continuous variables were analyzed using the Mann–Whitney *U* test, while categorical variables were compared using the Chi-square test or Fisher's exact test, as appropriate.

To identify age-associated microbial features within high-dimensional taxonomic profiles, we applied a two-step strategy. LEfSe (Linear Discriminant Analysis Effect Size; LDA > 2.0) (Segata et al., 2011) was first used for univariate screening. Multivariable association testing was then conducted using MaAsLin2 (Mallick et al., 2021). Age was modeled as a binary age-group variable (6 vs. 1 month) because the cohort included only two prespecified sampling timepoints, and this variable was designated as the primary variable of interest. The primary MaAsLin2 model adjusted for feeding type, delivery mode, maternal age, gestational weight gain, maternal pre-pregnancy BMI, parity, and maternal GDM, with infant identifier included as a random effect to account for repeated samples. Samples with missing feeding type data were excluded from the primary model, yielding a complete-case analytic set of 72 samples. The *p*-values were corrected using the Benjamini–Hochberg procedure and are reported as *q*-values. Given the sample size and the exploratory nature of candidate taxa identification, we defined *q* < 0.25 as the exploratory threshold and *q* < 0.05 as stringent evidence.

To assess robustness to missing feeding information, we repeated the MaAsLin2 model in all 82 samples by coding missing feeding type as a separate “Missing” category. Because infant sex showed a borderline imbalance between retained and non-retained infants, we additionally fitted a sex-adjusted full-cohort sensitivity model by including infant sex as an additional fixed effect. Because 28 infants provided samples at both 1 and 6 months, we also performed paired sensitivity analyses using two-sided Wilcoxon signed-rank tests for prespecified summary outcomes, including alpha diversity, *Enterobacteriaceae* relative abundance, antagonism indices, and CPM-normalized *bla* relative abundance.

Colonization-resistance proxy signals were summarized using three complementary log-ratio indices. These indices are log-transformed derivatives of the classical *Bifidobacterium*/*Enterobacteriaceae* (B/E) ratio, a commonly used ecological proxy for the balance between commensal-associated taxa and facultative pathobionts in early-life gut communities (Shao et al., 2024).

To accommodate zero abundances, a minimal pseudo-count (ε = 10^−5^) was uniformly added prior to transformation. This value was selected to avoid undefined logarithms while remaining below the observed non-zero abundance scale. To assess robustness to pseudo-count selection, we repeated the antagonism-index analyses using alternative values, including ε = 10^−4^ and the minimum observed non-zero abundance. The indices were defined as follows:


$$\begin{array}{l} AI_{1}=\log_{10}\frac{\text{Abundance of Bacteroides}+\varepsilon }{\text{Abundance of Enterobacteriaceae}+\varepsilon } \end{array}$$ (1)

$$\begin{array}{l} AI_{2}=\log_{10}\frac{\text{Relative abundance of Bifidobacterium}+\varepsilon }{\text{Relative abundance of Enterobacteriaceae}+\varepsilon } \end{array}$$ (2)

$$\begin{array}{l} AI_{3}=\log_{10}\frac{\text{Total beneficial taxa}+\varepsilon }{\text{Total pathobiont taxa}+\varepsilon } \end{array}$$ (3)

For *AI*_3_, taxa were grouped into prespecified ecological proxy categories. “Beneficial taxa" were operationally defined as the aggregate of five health-associated genera (*Bifidobacterium, Bacteroides, Lactobacillus, Faecalibacterium*, and *Roseburia*) (Rinninella et al., 2019). Conversely, “pathobiont taxa" comprised the family *Enterobacteriaceae* (Moreira de Gouveia et al., 2024) and the genus *Staphylococcus* (Bartlett et al., 2022). Taxa abundances were aggregated at the family and genus levels to mitigate sparsity and capture broad ecological patterns. This logarithmic transformation centers the indices around zero. Negative values indicate a relative abundance profile enriched for the denominator taxa, whereas positive values indicate a profile more consistent with colonization-resistance potential.

Finally, co-occurrence patterns between dominant bacterial taxa and ARGs were evaluated using Spearman's rank correlation, with effect sizes and *p* values reported for all tested associations. Correlations with |*r*|>0.6 were considered strong; associations with |*r*| between 0.3 and 0.6 were considered moderate and are reported with appropriate effect-size qualification. Visualizations were generated using the *ggplot2* and *pheatmap* packages in R.

## 3 Results

### 3.1 Age-associated shifts in gut microbiota diversity and community structure

To assess potential attrition bias, baseline characteristics were compared between the retained cohort (*n* = 28) and those lost to follow-up (*n* = 26). Most infant birth parameters and delivery modes were comparable between the retained and non-retained cohorts. However, attrition differed by parity (*p* = 0.009) and maternal GDM status (*p* = 0.037), while infant sex showed a borderline imbalance (67.9% male in the retained cohort vs. 38.5% in the non-retained cohort; *p* = 0.055; Table 1). Feeding type data were missing for seven baseline samples, including five retained and two non-retained infants. Across all 82 fecal samples, feeding type was missing for 10 samples: seven at C1 and three at C6.

**Table 1:** Cohort characteristics of study participants.

Characteristic	Retained cohort (*n* = 28)	Non-retained cohort (*n* = 26)	Test statistic	*p*-Value
Maternal factors				
Maternal age	30.26 ± 3.58	32.85 ± 6.04	*U* = 252.0	0.054
Gestational weight gain (kg)	14.56 ± 4.00	14.45 ± 4.13	*U* = 382.5	0.755
Pre-pregnancy BMI (kg/m^2^)	19.68 ± 2.25	21.48 ± 3.22	*U* = 254.5	0.059
GDM, *n* (%)	2 (7.1%)	8 (30.8%)	Fisher's exact	0.037^*^
GBS colonization, *n* (%)	2 (7.1%)	3 (11.5%)	Fisher's exact	0.663
Infant and delivery factors				
Gestational age (weeks)	39.55 ± 1.04	39.34 ± 0.88	*U* = 384.5	0.544
Birth weight (kg)	3.24 ± 0.28	3.40 ± 0.41	*U* = 276.5	0.132
Infant sex (Male), *n* (%)	19 (67.9%)	10 (38.5%)	Fisher's exact	0.055
Delivery mode (Vaginal), *n* (%)	22 (78.6%)	18 (69.2%)	Fisher's exact	0.540
Parity (Primiparous), *n* (%)	19 (67.9%)	7 (26.9%)	χ^2^ = 9.41	0.009^*^
Dietary factors				
Feeding type at sampling			χ^2^ = 3.48	0.175
Exclusive breastfeeding, *n* (%)	13 (56.5%)	15 (62.5%)		
Formula feeding, *n* (%)	5 (21.7%)	1 (4.2%)		
Mixed feeding, *n* (%)	5 (21.7%)	8 (33.3%)		

Data are presented as mean ± SD for continuous variables or *n* (%) for categorical variables. Baseline characteristics were compared between the retained cohort (*n* = 28) and the non-retained cohort (*n* = 26) to assess potential attrition bias. P-values were calculated using the Mann–Whitney *U* test for continuous variables and the Chi-square test (or Fisher's exact test when appropriate) for categorical variables. Significant differences (*p* < 0.05) are indicated with an asterisk (^*^). GDM: gestational diabetes mellitus; GBS: Group B Streptococcus. Feeding type data at baseline were unavailable for seven infants, including five retained and two non-retained infants; therefore, feeding-type percentages were calculated among infants with available baseline feeding data (retained cohort, *n* = 23; non-retained cohort, *n* = 24).

**Figure 1:**
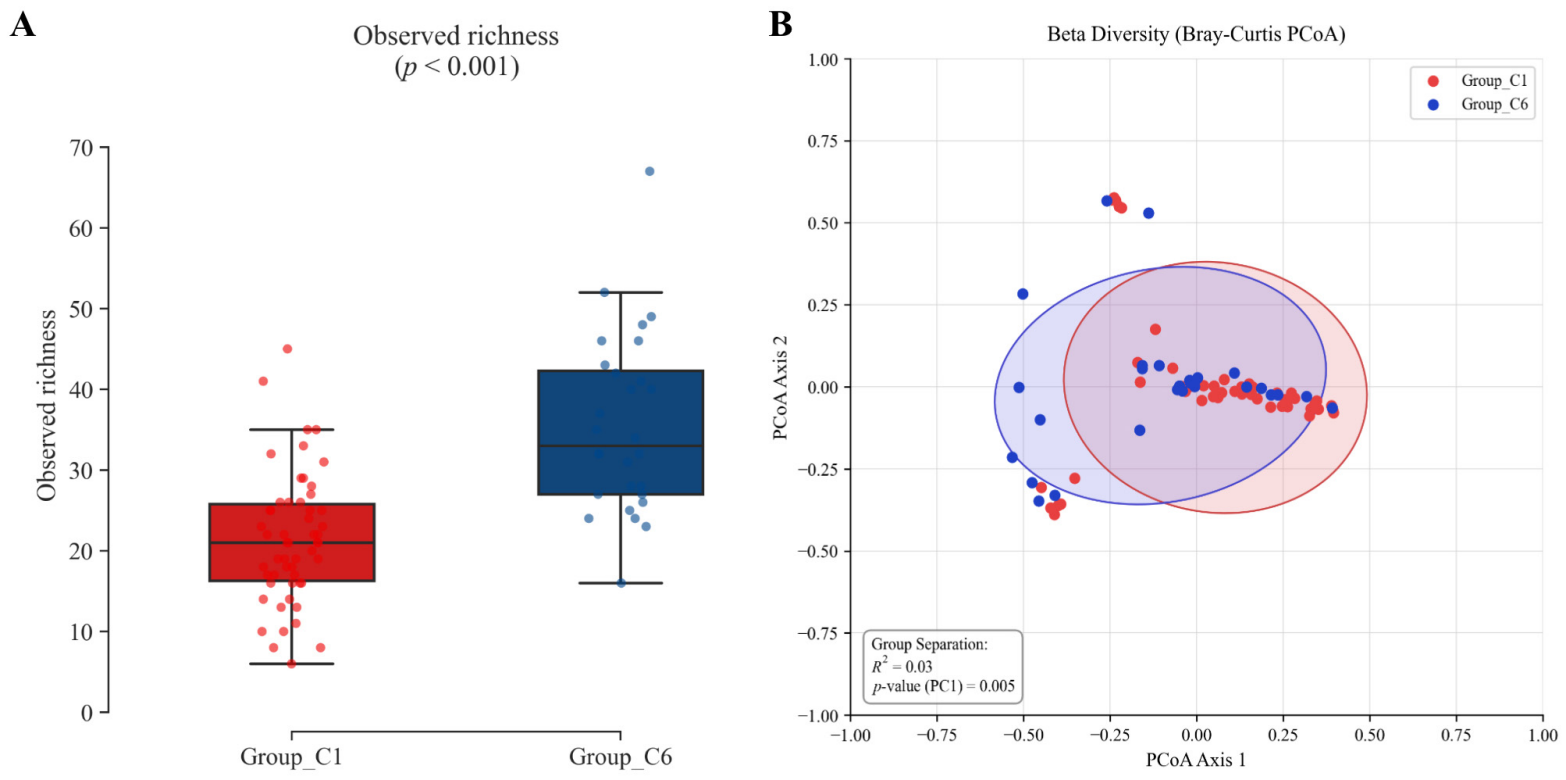
Age-associated Shifts in Gut Microbiota Diversity and Community Structure. **(A)** Boxplots of Alpha diversity measured by Observed Species richness. The 6-month group (C6) exhibits significantly higher species richness compared to the 1-month group (C1), indicating progressive colonization (*p* < 0.001, Wilcoxon rank-sum test). **(B)** Principal Coordinates Analysis (PCoA) based on Bray-Curtis dissimilarity. The distribution of C1 and C6 samples indicates age-associated differences in community structure (PERMANOVA *R*^2^ = 0.03, *p* = 0.005). Ellipses represent 95% confidence intervals.

Regarding community structure, we observed that species richness expanded markedly from 1 to 6 months (*p* < 0.001, Figure 1A), yet community evenness (Shannon/Simpson indices) remained stable (*p*>0.05, Supplementary Figure S1). Furthermore, principal coordinates analysis (PCoA) showed age-associated differences in community structure (PERMANOVA, *R*^2^ = 0.03, *p* = 0.005, Figure 1B).

### 3.2 Age-associated changes in taxonomic composition

**Figure 2:**
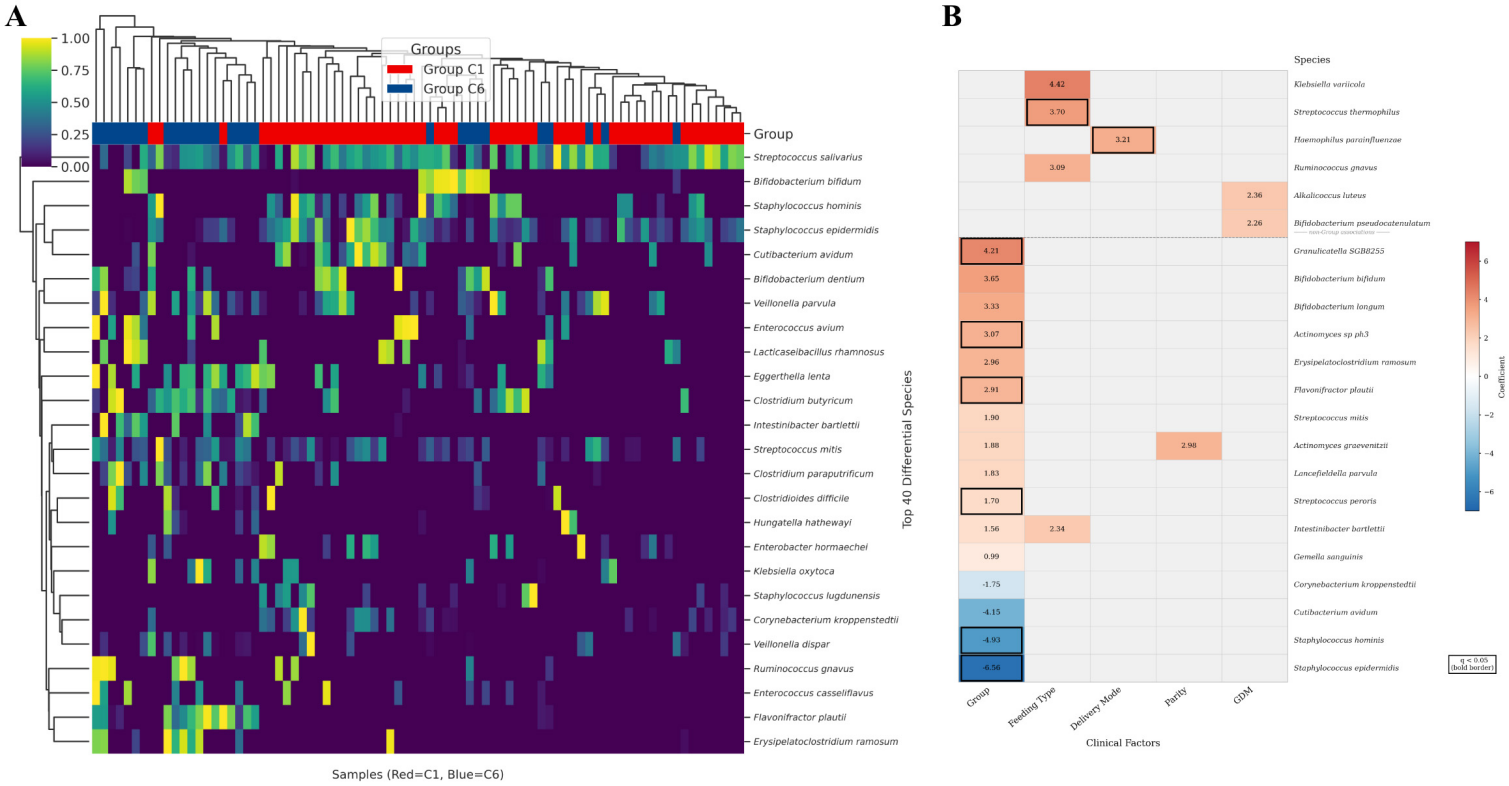
Taxonomic composition of infant gut microbiota at 1 and 6 months. **(A)** Heatmap showing the relative abundance of dominant species across all samples based on hierarchical clustering. **(B)** Multivariate association heatmap (MaAsLin2) identifying bacterial species significantly associated with clinical factors (age group, feeding type, delivery mode, gestational diabetes mellitus (GDM), and maternal age). The heatmap displays coefficients from the multivariate model. Features with *q* < 0.25 are displayed; significant associations (*q* < 0.05) are highlighted with bold borders.

Hierarchical clustering (Figure 2A) and prevalence analysis (Supplementary Figure S2) indicated that 1-month samples were characterized by higher relative abundance of aerotolerant pioneer taxa, whereas 6-month samples showed higher relative abundance of several anaerobic and oral-associated taxa.

To describe taxa associated with age group while accounting for measured clinical covariates, we applied multivariable MaAsLin2 modeling (Supplementary Table S1; Figure 2B) alongside univariate LEfSe analysis (Supplementary Figure S3). Skin-associated taxa were less abundant at 6 months than at 1 month. *Staphylococcus epidermidis* showed the strongest negative association with the 6-month group (coef = −6.56, FDR *q* < 0.001; Figure 2B, Supplementary Table S1), followed by *Staphylococcus hominis* (coef = −4.93, FDR *q* = 0.049) and *Cutibacterium avidum* (coef = −4.15, FDR *q* = 0.060). These associations were consistent in full-cohort sensitivity analyses (Supplementary Figures S4, S5).

By contrast, 6-month samples showed higher relative abundance of several strict anaerobic taxa. Multivariable modeling identified age-associated enrichment of *Bifidobacterium bifidum* (coef = 3.65, FDR *q* = 0.196) and *Bifidobacterium longum* (coef = 3.33, FDR *q* = 0.242), as well as obligate anaerobic specialists including *Flavonifractor plautii* (coef = 2.91, FDR *q* = 0.049) and *Actinomyces* sp. ph3 (coef = 3.07, FDR *q* < 0.001; Supplementary Table S1), independent of clinical covariates. *Ruminococcus gnavus* showed an exploratory association with formula feeding in the multivariable model (coef = 3.09, FDR *q* = 0.199) rather than with age group. In descriptive feeding-stratified analyses among samples with available feeding data (*n* = 72), *R. gnavus* was sparse in all groups (median relative abundance ≈0% in exclusively breastfed, mixed-fed, and formula-fed samples), with non-zero detection more frequent in formula-fed (four of 15, 26.7%) and mixed-fed (four of 19, 21.1%) than in exclusively breastfed samples (one of 38, 2.6%). *Granulicatella* SGB8255 was among the most statistically significant age-enriched taxa (coef = 4.21, FDR *q* < 0.001; Supplementary Table S1), a finding replicated in sensitivity analyses (Supplementary Table S1). Exploratory Spearman correlation analysis further identified a negative association between *Enterococcus faecium* abundance and birth weight (*r* = −0.40, *p* < 0.05; Supplementary Figure S6A).

These age-associated patterns were robust in sensitivity analyses (Supplementary Tables S5, S6). In the full-cohort model (*n* = 82), in which missing feeding type was coded as a separate category, 17 taxa were associated with age group at the exploratory threshold of *q* < 0.25 and 10 remained significant at *q* < 0.05, retaining the same direction of effect for the major C6-depleted taxa (e.g., *Staphylococcus epidermidis, Staphylococcus hominis, Cutibacterium avidum*) and C6-enriched taxa (e.g., *Granulicatella* SGB8255, *Flavonifractor plautii, Actinomyces* sp. ph3, *Bifidobacterium longum*, and *Bifidobacterium bifidum*). An additional sex-adjusted full-cohort model yielded the same age-associated patterns, and no taxon was associated with infant sex at the exploratory FDR threshold of *q* < 0.25 (minimum *q* = 0.491).

We also examined taxa not included in the antagonism indices but relevant to early-life gut ecology. *Veillonella* was detected in a subset of samples and was therefore described separately rather than assigned to either the beneficial or pathobiont proxy category. Clostridiales-related taxa showed heterogeneous patterns: *Flavonifractor plautii* and *Erysipelatoclostridium ramosum* were higher in 6-month samples in sensitivity analyses, whereas the broader Clostridiales group was not treated as a fixed colonization-resistance category.

### 3.3 Changes in pathobiont abundance and colonization resistance indices

**Figure 3:**
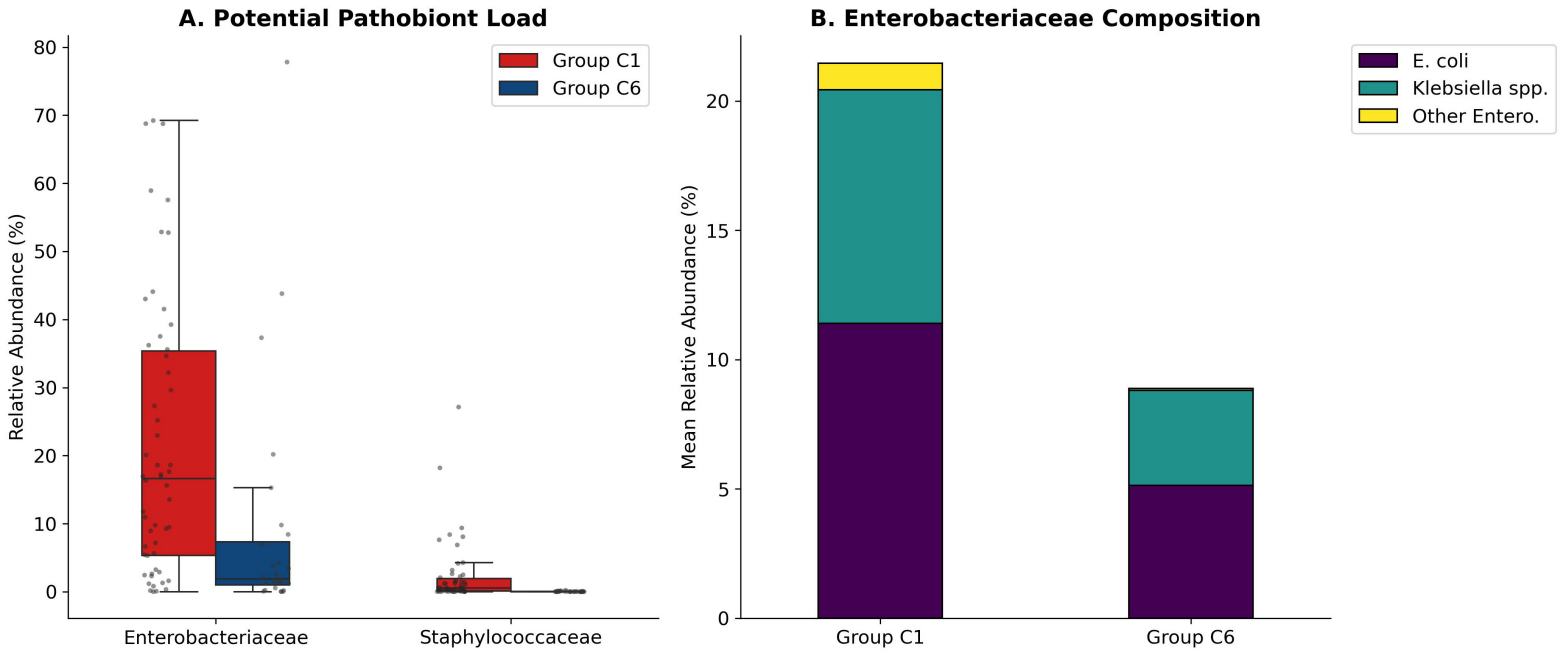
Dynamics of pathobiont-associated taxa and intra-family composition of *Enterobacteriaceae*. **(A)** Boxplots showing lower relative abundance of pathobiont-associated families at 6 months compared with 1 month. The median relative abundance of *Enterobacteriaceae* decreased from 16.64% (Q1,Q3: 5.33,35.35%) to 1.86% (Q1,Q3: 1.02,7.34%) (*p* < 0.001), and *Staphylococcaceae* decreased from 0.52% to 0.01% (*p* < 0.001). Detailed statistics are provided in Supplementary Table S2. **(B)** Stacked bar plots showing the mean genus-level composition within *Enterobacteriaceae*. Despite lower family-level *Enterobacteriaceae* relative abundance at 6 months, neither *Escherichia coli* (mean: 11.41 vs. 5.13%; *p* = 0.342) nor *Klebsiella* spp. (mean: 9.03 vs. 3.68%; *p* = 0.104) showed a statistically significant reduction.

**Figure 4:**
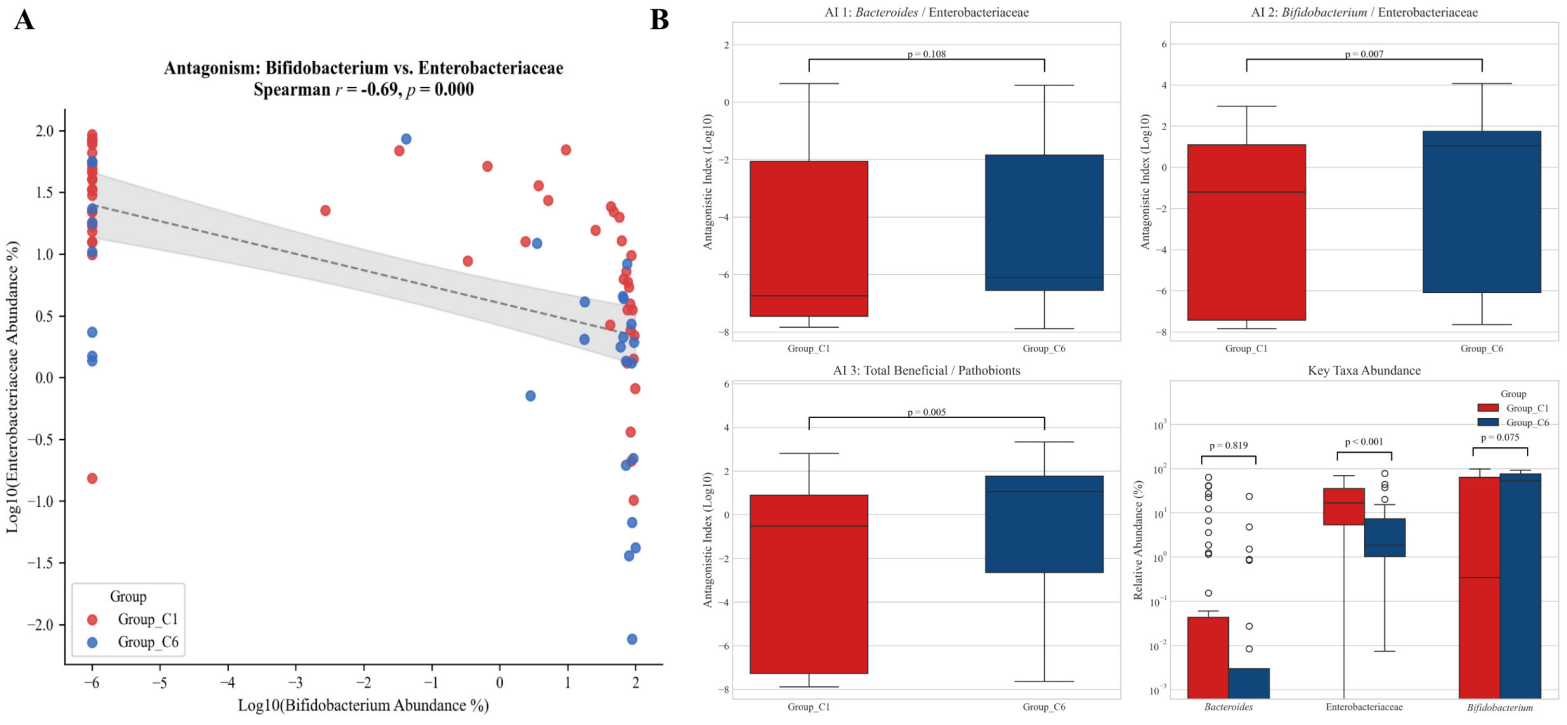
Establishment of colonization resistance via *Bifidobacterium*-mediated competitive exclusion. **(A)** Spearman correlation analysis showed a strong negative relationship (ρ = −0.69, *p* < 0.001) between the relative abundances of *Bifidobacterium* and *Enterobacteriaceae*. Each dot represents an individual infant sample. The gray shaded area indicates the 95% confidence interval of the regression line. **(B)** Evaluation of the Antagonistic Index (AI) and key taxa abundances. At the group level, the median AI for Bifidobacterium vs. Enterobacteriaceae (AI2) and total beneficial taxa vs. pathobionts (AI3) shifted from negative in the 1-month group (C1) to positive in the 6-month group (C6), marking a significant population-level transition (*p* = 0.007 and *p* = 0.005, respectively). In contrast, the AI for Bacteroides vs. Enterobacteriaceae (AI1) remained negative with no significant difference. The bottom-right panel displays the relative abundances of these key taxonomic drivers across the two age groups.

We examined whether age-associated taxonomic differences were accompanied by changes in pathobiont-associated bacterial groups (Figure 3; Supplementary Table S2). Figure 3A shows that *Enterobacteriaceae* relative abundance was lower at 6 months than at 1 month, decreasing from a median of 16.64%–1.86% (*p* < 0.001; Supplementary Table S2). *Staphylococcaceae* relative abundance also decreased, with the *Staphylococcus* genus declining from a median of 0.52%–0.01% (*p* < 0.001; Supplementary Table S2). Figure 3B further shows the genus-level composition within *Enterobacteriaceae*. Although family-level *Enterobacteriaceae* relative abundance was lower at 6 months, neither *Escherichia coli* nor *Klebsiella* spp. showed a statistically significant genus-level reduction (*E. coli*: *p* = 0.342; *Klebsiella* spp.: *p* = 0.104; Supplementary Table S2). This pattern suggests that the family-level decrease was not uniformly reflected across all constituent genera. We then assessed colonization-resistance proxy patterns using taxon–taxon antagonism metrics (Equations 1–3). Spearman correlation analysis showed a strong negative relationship (ρ = −0.69, *p* < 0.001) between the relative abundances of *Bifidobacterium* and *Enterobacteriaceae* (Figure 4A). At the population level, both *AI*_2_ and *AI*_3_ increased significantly from 1 to 6 months (Mann–Whitney *U*, *p* = 0.007 and *p* = 0.005, respectively; Figure 4B; Supplementary Table S3). This shift was further supported by paired analyses restricted to the 28 longitudinal infant pairs (both *p* < 0.01; Table 2). Stratified analyses showed no statistically significant differences in AI scores across delivery modes, feeding types, or maternal clinical factors (Supplementary Figures S6B–F; *p*>0.05). The *AI*_2_ and *AI*_3_ findings were robust to the choice of pseudo-count, retaining the same direction and statistical support under ε = 10^−4^ and the minimum observed non-zero abundance (Supplementary Table S3).

**Table 2:** Sensitivity analysis using paired comparisons in infants with longitudinal samples (*n* = 28 pairs): alpha diversity, *Enterobacteriaceae*, antagonism indices (AI), and CPM-normalized β-lactamase (*bla*) relative abundance.

Outcome	C1 (1 month), median (Q1, Q3)	C6 (6 months), median (Q1, Q3)	Paired Wilcoxon *p*
Observed richness	21.0 (16.0, 25.0)	33.0 (27.0, 42.3)	<0.001
Shannon diversity	1.47 (0.90, 1.63)	1.40 (1.14, 1.70)	0.186
*Enterobacteriaceae* relative abundance	17.09 (5.39, 27.87)	1.86 (1.02, 7.34)	0.002
AI2 (Bifidobacterium vs. Enterobacteriaceae)	−1.20 (−7.41, 1.03)	1.05 (−6.09, 1.75)	0.007
AI3 (Total beneficial vs. pathobiont)	−0.53 (−7.30, 0.68)	1.05 (−2.64, 1.77)	0.002
CPM-normalized β-lactamase (*bla*) relative abundance	8.59 × 10^−5^ (5.21 × 10^−5^, 1.56 × 10^−4^)	1.26 × 10^−4^ (4.48 × 10^−5^, 1.75 × 10^−4^)	0.465

### 3.4 Age-associated changes in metabolic pathways

**Figure 5:**
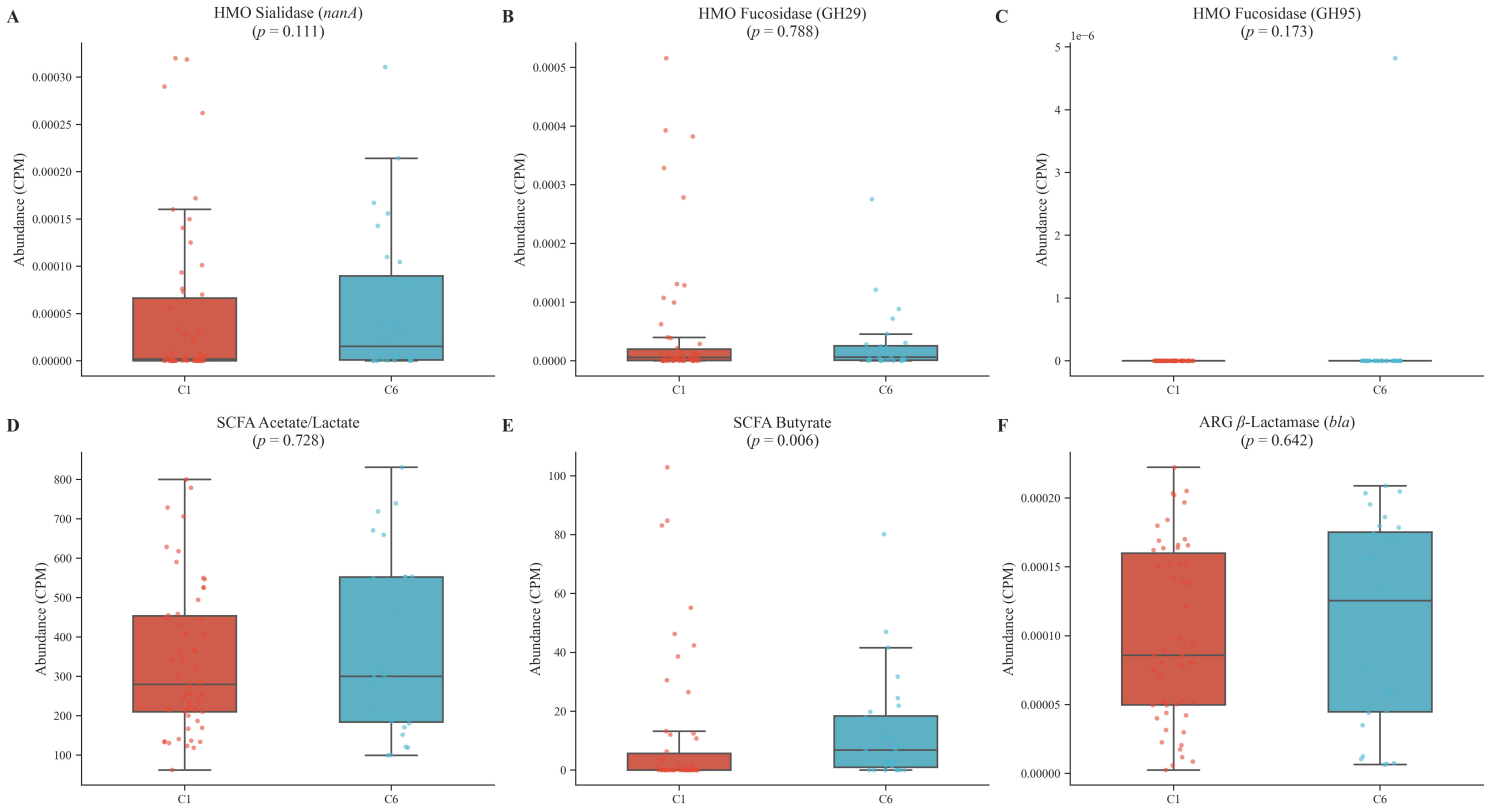
Age-associated differences in selected functional pathway abundances. Functional profiling (HUMAnN3) showed selected age-associated differences in short-chain fatty acid (SCFA)-related pathway abundance between 1 and 6 months. **(A)** HMO sialidase (*nanA*), **(B)** HMO fucosidase GH29, **(C)** HMO fucosidase GH95, and **(D)** SCFA acetate/lactate production pathways showed no statistically significant differences between C1 and C6 groups (all *p*>0.05). **(E)** Butyrate biosynthesis pathway abundance was higher in the C6 group (*p* = 0.006, Mann–Whitney *U* test), consistent with a community-level functional shift toward anaerobic fermentation potential. **(F)** CPM-normalized β-lactamase (*bla*) relative abundance showed no statistically significant difference between timepoints (*p* = 0.642). Center lines represent medians; box limits indicate the 25th and 75th percentiles. CPM, copies per million.

Taxonomic shifts occurred alongside targeted functional changes rather than a global metabolic turnover (Figure 5). Broad metabolic functions related to HMO degradation, including sialidase *nanA*, fucosidase GH29, and GH95, as well as acetate and lactate production, remained stable between timepoints (Figures 5A–D; all *p*>0.05). In contrast, butyrate biosynthesis pathway abundance was undetectable in the majority of 1-month infants (median = 0; Q1,Q3: 0, 5.60 CPM), rising to 6.79 CPM (Q1,Q3: 0.91, 18.33) at 6 months (*p* = 0.006; Figure 5E; Supplementary Table S4). This functional pattern occurred alongside the age-associated expansion of several anaerobic taxa, including *Flavonifractor plautii* (MaAsLin2 coef = 2.91, FDR *q* = 0.049; Supplementary Table S1); however, this association should not be interpreted as direct evidence that *F. plautii* accounted for the butyrate biosynthesis signal.

Exploratory taxon–function correlation analysis further identified a negative association between *Haemophilus parainfluenzae* and sialidase *nanA* at 6 months (ρ = −0.53, *p* < 0.01; Supplementary Figure S7). This association is reported descriptively and should not be interpreted as evidence of a direct functional interaction.

### 3.5 Changes in *bla* gene abundance and associations with gut microbiota composition

As described above, the family-level decline in *Enterobacteriaceae* relative abundance was not mirrored by significant genus-level reductions in *Escherichia coli* or *Klebsiella* spp. (Figure 3B). Against this background of persistent constituent genera, we examined the β-lactamase reservoir. Of the three ARG classes profiled, tetracycline efflux and vancomycin resistance (*vanA*) genes were undetected across all 82 samples, and total CPM-normalized *bla* relative abundance showed no statistically significant difference between timepoints (*p* = 0.642; Figure 5F; Supplementary Table S4).

Spearman correlation analysis revealed distinct association patterns between *bla* relative abundance and microbial composition at each timepoint (Supplementary Figure S7). At 1 month, *Bifidobacterium pseudocatenulatum* showed the strongest positive correlation with *bla* (ρ = 0.53, *p* < 0.001), whereas *Escherichia coli* was negatively correlated with *bla* (ρ = −0.65, *p* < 0.001). At 6 months, *Bifidobacterium bifidum* emerged as the strongest positive correlate of *bla* (ρ = 0.54, *p* < 0.01). Additional significant exploratory correlations are shown in Supplementary Figure S7.

### 3.6 Genome-resolved host attribution of *bla* genes

To attribute the detected resistance genes to specific genomes, we recovered 171 MAGs from the cohort (median completeness 97.9%, median contamination 0.47%; 112 near-complete at ≥90% completeness and ≤ 5% contamination), spanning six bacterial phyla (Supplementary Figure S8; Supplementary Table S8). These included eight high-quality *Bifidobacterium* MAGs (*B. infantis, B. longum, B. bifidum, B. breve, B. pseudocatenulatum, B. dentium, B. adolescentis*, and *B. animalis*) as well as MAGs of the principal pathobiont taxa (*Klebsiella* spp., *Escherichia coli, Enterobacter, Serratia*, and *Staphylococcus* spp.).

RGI/CARD annotation of the MAGs assigned all 15 hydrolytic β-lactamase gene hits to *Enterobacteriaceae* (*Klebsiella*: SHV-71, LEN-16, CMY-190; *E. coli*: CMY-189, EC-8; *Enterobacter hormaechei*: ACT-45; *Serratia ureilytica*: SRT-3), *Staphylococcus* (BlaZ), and *Bacteroides* (CblA-1, CepA-44, CfiA4, CfxA3) / *Dysgonomonas* (DYB-1) MAGs (Supplementary Figure S9; Supplementary Table S9). No β-lactamase gene was detected in any of the eight *Bifidobacterium* MAGs, which instead carried non-β-lactam determinants (tet(W), tet(O), and intrinsic rpoB and ileS variants). The detected β-lactamases were predominantly intrinsic, species-associated enzymes (AmpC-type ACT/CMY/EC/SRT and class A SHV/LEN in *Enterobacteriaceae*, intrinsic *Bacteroides* enzymes, and staphylococcal BlaZ); the classic mobile families *bla*_*TEM*_ and *bla*_*OXA*_ were not detected, and a single *bla*_*CTX*−*M*_ hit (CTX-M-14) occurred on an unplaced contig within a Firmicutes bin, most consistent with misbinning of a mobile element. Penicillin-binding protein (PBP3) target mutations (*n* = 14) and porin/efflux determinants were detected separately and are not hydrolytic β-lactamases.

geNomad classified 930 contigs as plasmids and 675 as proviruses across the MAG catalog. Cross-referencing the 15 hydrolytic β-lactamase genes against these predictions, 4 were located on contigs classified as plasmids, all within *Enterobacteriaceae* MAGs: *Klebsiella* (SHV-71, LEN-16, and CMY-190) and *Serratia ureilytica* (SRT-3) (plasmid score ≥0.93; Supplementary Table S10), although the LEN-16-carrying contig was atypically large (477 kb, non-circular) and its plasmid assignment is therefore less certain. The remaining genes—including the intrinsic *Bacteroides* (CblA-1, CepA-44, CfiA4, CfxA3) and *Dysgonomonas* (DYB-1) enzymes, staphylococcal BlaZ, and the residual *Enterobacteriaceae* enzymes of *Escherichia coli* (EC-8, CMY-189) and *Enterobacter hormaechei* (ACT-45)—were located on non-plasmid contigs consistent with a chromosomal context. No β-lactamase gene was located on a predicted proviral contig, and the single CTX-M-14 hit remained on a short, unplaced contig that was not classified as a plasmid. Conjugation hallmark genes were not detected on the four *bla*-bearing plasmid contigs (Supplementary Table S10).

## 4 Discussion

This study provides a multivariable-adjusted characterization of early infant gut microbiome succession in an antibiotic-restricted prospective cohort. Our primary goal was to describe age-group-associated microbiome and relative resistome patterns between 1 and 6 months while accounting for measured perinatal and maternal covariates. Compared with 1-month samples, microbiome profiles at 6 months were characterized by lower relative abundance of aerotolerant pioneer taxa, higher relative abundance of several anaerobic taxa, and higher colonization-resistance proxy indices. By contrast, CPM-normalized β-lactamase (*bla*) relative abundance showed no statistically significant timepoint difference. We did not detect statistically significant heterogeneity in colonization-resistance proxy indices across delivery mode, feeding type, or maternal clinical strata. Given the modest sample size and limited exposure contrast, these null findings should not be interpreted as evidence that such factors are unimportant in infant microbiome assembly. Rather, they suggest that age-associated ecological patterns remained detectable in this antibiotic-restricted cohort after adjustment for measured covariates.

The age-associated displacement of skin- and oral-associated pioneers is consistent with canonical foundational succession documented in prior longitudinal cohort studies (Bäckhed et al., 2015). The taxa most prominently involved include *Staphylococcus epidermidis, Staphylococcus hominis*, and *Cutibacterium avidum*. Beyond this canonical pattern, *Granulicatella* SGB8255 and *Actinomyces* sp. ph3 were both significantly enriched at 6 months. This enrichment is consistent with oral-to-gut seeding associated with complementary feeding initiation (Kunath et al., 2024; Dewhirst et al., 2010). These oral-origin signals underscore that microbiome maturation between 1 and 6 months encompasses not only canonical anaerobic succession but also the progressive acquisition of commensals whose ecological roles in the infant gut remain poorly defined.

The multivariable associations of *Bifidobacterium bifidum* and *Flavonifractor plautii* with the 6-month ecosystem are consistent with the emergence of a keystone anaerobic community. Early facultative colonizers are thought to deplete luminal oxygen progressively. This generates redox conditions that selectively favor strict anaerobic taxa (Albenberg et al., 2014; Litvak et al., 2018). In our covariate-adjusted model, *Ruminococcus gnavus* showed an exploratory association with formula feeding rather than age group. Descriptive analyses indicated sparse detection overall, with non-zero detection more frequent in formula-fed and mixed-fed samples than in exclusively breastfed samples. This finding should be interpreted cautiously given the modest subgroup sizes and zero-inflated abundance distribution, but it supports the inclusion of feeding type as a covariate in microbiome succession analyses.

The apparent uniformity of colonization-resistance indices across delivery modes and feeding strata warrants careful ecological interpretation. Contemporary priority-effects models suggest that early *Bifidobacterium* dominance exerts powerful niche-preemption effects on subsequent community assembly (Shao et al., 2024). The ecological resilience observed here may partly reflect luminal acidification, HMO-driven metabolic niche occupation, and spatial exclusion collectively buffering community composition against the influence of delivery mode. This buffering is not unconditional, however. Cohorts with greater exposure contrast have shown that cesarean delivery, early breastfeeding cessation, and peripartum antibiotic exposure can delay bifidobacterial establishment and attenuate colonization-resistance features (Shao et al., 2024; Selma-Royo et al., 2024; Reyman et al., 2022; Samarra et al., 2025). The resilience observed here therefore likely reflects the favorable colonization conditions that this antibiotic-restricted, predominantly breastfed cohort was specifically designed to capture.

Colonization resistance in early infancy may develop through sequential ecological and metabolic changes. The upstream barrier may depend on bifidobacterial fermentation of HMOs to acetate and lactate, driving luminal acidification and reducing colonization permissibility for *Enterobacteriaceae* (Laursen and Roager, 2023; Ojima et al., 2022). *Bifidobacterium* strains harboring the *aldh* gene further convert aromatic amino acid into phenyllactic acid, 4-OH-PLA, and ILA, which suppress infant-derived *E. coli, K. pneumoniae*, and *C. freundii* via pH-independent mechanisms (Chatzigiannidou et al., 2025). The downstream barrier may emerge concomitantly with the expansion of butyrate biosynthetic capacity. Colonocyte oxidation of butyrate depletes oxygen at the mucosal interface. This oxygen gradient excludes facultative anaerobes from the epithelial surface (Albenberg et al., 2014; Litvak et al., 2018). These mechanisms are supported by prior experimental work but were beyond the scope of this study.

The stability of HMO-related enzyme markers (sialidase *nanA* and fucosidases GH29 and GH95) between 1 and 6 months may partly reflect continued breast milk exposure in a substantial proportion of infants at the later timepoint. However, because feeding type was recorded categorically and the proportion of breast milk vs. formula among mixed-fed infants was not collected, we could not evaluate dose-response relationships between breast milk exposure and HMO-related functional markers.

The absence of a statistically significant difference in CPM-normalized *bla* relative abundance despite lower *Enterobacteriaceae* relative abundance requires cautious interpretation. Because shotgun metagenomic outputs were analyzed as relative abundance profiles, the expansion of obligate anaerobes between 1 and 6 months changes the compositional denominator against which ARG signals are measured. Therefore, stable CPM-normalized *bla* relative abundance cannot determine whether absolute *bla* copy number per gram of feces remained stable, decreased, or increased. Absolute quantification using qPCR, spike-in standards, flow cytometry, or synthetic internal standards would be required to distinguish true changes in ARG burden from relative compositional shifts. External quantitative data further suggest that absolute ARG abundance may peak around 6 months before declining toward adult levels, underscoring the need to separate relative resistome profiles from absolute ARG trajectories (Chatzigiannidou et al., 2025).

The positive correlations between CPM-normalized *bla* relative abundance and selected *Bifidobacterium* species should also be interpreted cautiously. These species-level associations are compatible with a *Bifidobacterium*-associated *bla* signal, but they may also partly reflect compositional coupling in relative-abundance data as *Bifidobacterium* expands and *Enterobacteriaceae* contracts proportionally. Although intrinsic *bla* genes have been reported in some infant gut-associated *Bifidobacterium* strains, including *B. longum* subsp. *infantis* (Fouhy et al., 2013; Taft et al., 2018), we did not detect a β-lactamase gene in any of the eight *Bifidobacterium* MAGs in this cohort. Recent work has further linked *Bifidobacterium*-dominated early-life communities with infant resistome development and antibiotic resistance acquisition (Samarra et al., 2024, 2025), while studies of neonatal microbiota assembly support a role for *Bifidobacterium* in pathogen colonization resistance (Shao et al., 2024). Consistent with these reports, MAG-resolved annotation assigned all detected β-lactamase genes to residual *Enterobacteriaceae* (*Klebsiella, Escherichia coli, Enterobacter*, and *Serratia*), *Staphylococcus*, and *Bacteroides*, whereas none of the *Bifidobacterium* MAGs carried a β-lactamase gene. This indicates that the positive *Bifidobacterium*–*bla* relative-abundance correlation reflects compositional coupling rather than genomic carriage by *Bifidobacterium*. As detailed in the Results, the detected enzymes were predominantly intrinsic, species-associated β-lactamases, with the classic mobile families largely absent and only a subset of residual *Enterobacteriaceae* enzymes localized to predicted plasmid contigs (Supplementary Table S10). Because plasmid prediction from short-read contigs cannot reconstruct complete, closed plasmids or confirm active conjugative transfer—and conjugation hallmark genes were not detected on the *bla*-bearing plasmid contigs—horizontal transfer could not be quantified; nonetheless, these data indicate that part of the residual *Enterobacteriaceae* β-lactam resistance pool is plasmid-associated and therefore potentially mobile (Chatzigiannidou et al., 2025).

Several methodological considerations should be noted. First, this study used relative metagenomic profiles rather than absolute bacterial load or absolute ARG copy number. The resistome results should therefore be interpreted as CPM-normalized relative abundance patterns, and future work using qPCR, spike-in standards, or quantitative metagenomics would help quantify absolute ARG burden. Second, 26 of 54 enrolled infants did not contribute samples at 6 months, limiting paired analyses to 28 dyads. With 28 longitudinal pairs, paired sensitivity analyses were adequately powered only to detect large standardized effects (*d*_*z*_≥0.55); moderate or small true differences in CPM-normalized *bla* relative abundance and Shannon evenness cannot be excluded on the basis of the current data. Retained and non-retained infants differed by parity and maternal GDM status, and infant sex showed a borderline imbalance; however, adjustment for parity and GDM, together with full-cohort and sex-adjusted sensitivity analyses, yielded consistent age-associated taxonomic patterns. Third, feeding type was missing for 10 of 82 samples and was recorded categorically among available samples. The primary MaAsLin2 model used the complete-case analytic set (*n* = 72), whereas sensitivity analyses retained all samples by coding missing feeding type as a separate category. More detailed feeding data, including the proportion of breast milk vs. formula and direct vs. expressed breast milk, would allow finer assessment of diet-related effects. Maternal postpartum antibiotic use was also not systematically documented. Unmeasured environmental exposures, including household pets, living conditions, sibling contact, daycare attendance, and maternal diet, were not collected and could not be evaluated as covariates. Fourth, although MAG-resolved annotation attributed the detected β-lactamase genes to specific host genomes (residual *Enterobacteriaceae, Staphylococcus*, and *Bacteroides* rather than *Bifidobacterium*), this attribution was restricted to binned contigs and therefore represents a subset of the read-based *bla* signal. Plasmid context was inferred from geNomad classification of short-read contigs rather than from complete, closed plasmid sequences; long-read sequencing and replicon typing would be required to confirm plasmid completeness and to establish conjugative transfer potential. Finally, this was a single-site cohort from an urban academic hospital with stringent infant antibiotic-restriction criteria. Recruitment occurred between November 2020 and October 2021, during the COVID-19 pandemic. Pandemic-related changes in healthcare-seeking behavior, hygiene practices, household contact patterns, and infant feeding routines may have influenced early-life microbial exposures and could not be directly evaluated. The findings are therefore most directly generalizable to infants with similarly low documented systemic antibiotic exposure.

These findings have two practical implications. The robustness of age-associated colonization-resistance signatures in this low-antibiotic-pressure cohort suggests that the infant gut possesses intrinsic ecological resilience, potentially mediated by the priority effects of *Bifidobacterium*-dominant pioneer communities. Strategies that promote early establishment of *aldh*-positive *Bifidobacterium* strains, such as exclusive breastfeeding promotion and synbiotic supplementation, could support colonization resistance in higher-risk infants, though direct intervention trial evidence is needed (Shao et al., 2024; Chatzigiannidou et al., 2025; Samarra et al., 2025). Conversely, the decoupling between ecological maturation and resistome attenuation points to a potential inadequacy in surveillance paradigms that rely solely on taxonomic endpoints as proxies for AMR burden. Resistome mitigation may require targeting the mobile gene pool through reduced antibiotic selection pressure and preservation of mucosal barrier integrity. Taxonomic maturation should not be equated with resistome attenuation. Future intervention trials and surveillance programs should therefore incorporate absolute ARG abundance and mobilome-aware approaches.

## Data Availability

The raw data generated in this study can be found in the CNGBdb, accession number CNP0009251 (https://doi.org/10.26036/CNP0009251).
