# Supplementary material for: Age-associated gut microbiome succession, colonization resistance, and relative resistome patterns in an antibiotic-restricted infant cohort
Source: Front Microbiol. 2026 Jul 8;17:1862116. doi: 10.3389/fmicb.2026.1862116 (PMC13388750; doi:10.3389/fmicb.2026.1862116)
Supplement: Supplementary file 1 [file Data_Sheet_1.pdf]

Supplementary Material

1 SUPPLEMENTARY TABLES

Table S1. Significant multivariable association statistics from the primary MaAsLin2 model (FDR < 0.25).

Feature	Metadata	Value	Coef	Stderr	Total Samples	Non-zero Samples	p	q
Staphylococcus epidermidis	group	C6	−6.56	0.72	72	53	<0.001	<0.001
Granulicatella SGB8255	group	C6	4.21	0.68	72	16	<0.001	<0.001
Actinomyces sp. ph3	group	C6	3.07	0.60	72	11	<0.001	<0.001
Streptococcus peroris	group	C6	1.70	0.41	72	8	<0.001	0.019
Streptococcus thermophilus	Feeding Type	Formula	3.70	0.99	72	25	<0.001	0.049
Flavonifractor plautii	group	C6	2.91	0.75	72	15	<0.001	0.049
Staphylococcus hominis	group	C6	−4.93	1.24	72	27	<0.001	0.049
Haemophilus parainfluenzae	Delivery Mode	C-Section	3.21	0.88	72	17	<0.001	0.049
Actinomyces graevenitzii	group	C6	1.88	0.53	72	12	<0.001	0.059
Cutibacterium avidum	group	C6	−4.15	1.13	72	21	<0.001	0.060
Intestinibacter bartlettii	Feeding Type	Formula	2.34	0.68	72	8	0.001	0.071
Gemella sanguinis	group	C6	0.99	0.31	72	10	0.003	0.152
Intestinibacter bartlettii	group	C6	1.56	0.51	72	8	0.003	0.187
Bifidobacterium bifidum	group	C6	3.65	1.19	72	14	0.004	0.196
Actinomyces graevenitzii	Parity	2	2.98	1.00	72	12	0.004	0.196
Alkalicoccus luteus	GDM	1	2.36	0.80	72	37	0.005	0.196
Corynebacterium kroppenstedtii	group	C6	−1.75	0.58	72	19	0.005	0.196
Streptococcus mitis	group	C6	1.90	0.63	72	40	0.005	0.196
Lancefieldella parvula	group	C6	1.83	0.62	72	12	0.005	0.196
Klebsiella variicola	Feeding Type	Mixed	4.42	1.56	72	17	0.006	0.199
Ruminococcus gnavus	Feeding Type	Formula	3.09	1.09	72	9	0.006	0.199
Erysipelatoclostridium ramosum	group	C6	2.96	1.04	72	10	0.006	0.199
Bifidobacterium longum	group	C6	3.33	1.17	72	18	0.008	0.242
Bifidobacterium pseudocatenulatum	GDM	1	2.26	0.82	72	8	0.009	0.248

Note: Total Samples represents the number of samples included in the MaAsLin2 multivariable model (analytic $n = 72$, excluding 10 samples with missing feeding type data). Non-zero Samples indicates the number of samples where the specific taxon was detected (abundance > 0). Species with stringent significance (FDR < 0.05) are visualized in Figure 2 of the main text. An exploratory threshold of FDR < 0.25 was applied to capture potentially relevant biological associations for hypothesis generation.

Table S2. Pathobiont abundance between 1-month and 6-month infants.

Pathobiont / Taxa	C1 Median (Q1, Q3)	C6 Median (Q1, Q3)	p
Enterobacteriaceae	16.64 (5.33, 35.35)	1.86 (1.02, 7.34)	<0.001
E. coli	5.02 (0.09, 17.00)	1.38 (0.46, 4.57)	0.342
Klebsiella spp.	2.57 (0.00, 10.08)	0.20 (0.01, 0.40)	0.104
Staphylococcaceae	0.52 (0.12, 1.95)	0.01 (0.00, 0.04)	<0.001

Note: Values are reported as median (Q1, Q3) relative abundance (%). p-values were calculated using the Mann–Whitney U test. Non-normal distribution confirmed for all taxa (Shapiro–Wilk $p < 0.05$). Mean relative abundance values for Enterobacteriaceae compositional breakdown are shown in Figure 3B of the main text.

Table S3. Antagonism indices and selected key bacterial taxa abundance between 1-month and 6-month infants.

Index / Taxa Name	C1 Median (Q1, Q3)	C6 Median (Q1, Q3)	p
Antagonism indices ($\log_{10}$ ratio)			
AI1 (Bacteroides vs. Enterobacteriaceae)	−5.74 (−6.46, −2.07)	−5.11 (−5.56, −1.84)	0.104
AI2 (Bifidobacterium vs. Enterobacteriaceae)	−1.20 (−6.43, 1.09)	1.05 (−5.09, 1.75)	0.007
AI3 (Total beneficial taxa vs. pathobiont taxa)	−0.53 (−6.42, 0.90)	1.05 (−3.75, 1.76)	0.005
Selected key taxa relative abundance (%)			
Bifidobacterium genus	0.34 (0.00, 63.09)	52.72 (0.00, 75.48)	0.075
Bacteroides genus	0.00 (0.00, 0.04)	0.00 (0.00, 0.00)	0.819

Note: p-values were calculated using the Mann–Whitney U test. Antagonism indices were calculated as $\log_{10}$ ratios after adding a pseudo-count of $\varepsilon = 10^{-5}$ to accommodate zero abundances. AI1 was defined as Bacteroides/Enterobacteriaceae, AI2 as Bifidobacterium/Enterobacteriaceae, and AI3 as total beneficial taxa/pathobiont taxa. Higher values indicate a relative abundance profile more consistent with colonization-resistance potential. Pseudo-count sensitivity analyses using $\varepsilon = 10^{-4}$ and the minimum observed non-zero abundance yielded unchanged conclusions for AI2 and AI3 (AI2: $p = 0.007$ under both settings; AI3: $p = 0.005$ under both settings), while AI1 remained non-significant.

Table S4. Functional pathway abundance between 1-month and 6-month infants.

Functional Marker	C1 Median (Q1, Q3)	C6 Median (Q1, Q3)	p
HMO Sialidase (nanA)	1.83×10^{-6} $(1.39\times10^{-7}, 6.64\times10^{-5})$	1.53×10^{-5} $(1.01\times10^{-6}, 8.99\times10^{-5})$	0.111
HMO Fucosidase (GH29)	5.80×10^{-6} $(6.11\times10^{-7}, 2.00\times10^{-5})$	6.16×10^{-6} $(8.79\times10^{-7}, 2.54\times10^{-5})$	0.788
HMO Fucosidase (GH95)	0 $(0, 0)$	0 $(0, 1.72\times10^{-7})$	0.173
CPM-normalized β-lactamase (bla) relative abundance*	8.59×10^{-5} $(4.97\times10^{-5}, 1.60\times10^{-4})$	1.26×10^{-4} $(4.48\times10^{-5}, 1.75\times10^{-4})$	0.642
SCFA Acetate/Lactate	279.26 (209.84, 453.53)	299.84 (184.00, 552.25)	0.728
SCFA Butyrate	0 $(0, 5.60)$	6.79 $(0.91, 18.33)$	0.006

Note: Values are reported as median (Q1, Q3). Units: CPM/RPK. p-values were calculated using the Mann–Whitney U test (all C1 samples, $n = 54$; C6 samples, $n = 28$).

* IQR estimates differ slightly from Table 2 of the main text, which was based on the 28 paired infants only (Mann–Whitney U replaced by paired Wilcoxon signed-rank test for that sensitivity analysis).

Table S5. Age-associated taxa from the full-cohort MaAsLin2 sensitivity analysis with missing feeding type coded as a separate category ($n = 82$).

Taxon	Direction at 6 months	Coefficient	q value
Granulicatella SGB8255	Higher	4.510	< 0.001
Staphylococcus epidermidis	Lower	-5.950	< 0.001
Flavonifractor plautii	Higher	3.620	< 0.001
Actinomyces sp. ph3	Higher	3.070	< 0.001
Intestinibacter bartlettii	Higher	2.410	0.003
Staphylococcus hominis	Lower	-5.190	0.005
Gemella sanguinis	Higher	1.700	0.005
Streptococcus peroris	Higher	1.700	0.005
Actinomyces graevenitzii	Higher	1.940	0.019
Streptococcus mitis	Higher	2.160	0.025
Lancefieldella parvula	Higher	1.910	0.063
Cutibacterium avidum	Lower	-3.650	0.075
Erysipelatoclostridium ramosum	Higher	3.030	0.088
Eggerthella lenta	Higher	2.680	0.108
Bifidobacterium longum	Higher	3.430	0.112
Corynebacterium kroppenstedtii	Lower	-1.690	0.112
Bifidobacterium bifidum	Higher	3.180	0.125

Note: Missing feeding type was coded as a separate "Missing" category to retain all 82 samples. Direction refers to relative abundance at 6 months compared with 1 month. Only age-associated taxa meeting the exploratory threshold of $q < 0.25$ are shown. Coefficients are shown to three decimal places; q values below 0.001 are shown as < 0.001.

Table S6. Age-associated taxa from the sex-adjusted full-cohort MaAsLin2 sensitivity analysis ($n = 82$).

Taxon	Direction at 6 months	Coefficient	q value
Granulicatella SGB8255	Higher	4.436	< 0.001
Staphylococcus epidermidis	Lower	-5.913	< 0.001
Flavonifractor plautii	Higher	3.654	< 0.001
Actinomyces sp. ph3	Higher	3.025	0.001
Intestinibacter bartlettii	Higher	2.390	0.005
Gemella sanguinis	Higher	1.707	0.007
Streptococcus peroris	Higher	1.666	0.009
Staphylococcus hominis	Lower	-5.149	0.019
Actinomyces graevenitzii	Higher	1.963	0.023
Streptococcus mitis	Higher	2.132	0.032
Cutibacterium avidum	Lower	-3.694	0.079
Lancefieldella parvula	Higher	1.885	0.081
Bifidobacterium longum	Higher	3.477	0.122
Corynebacterium kroppenstedtii	Lower	-1.705	0.122
Eggerthella lenta	Higher	2.630	0.122
Erysipelatoclostridium ramosum	Higher	2.944	0.122
Bifidobacterium bifidum	Higher	3.272	0.123

Note: Missing feeding type was coded as a separate "Missing" category to retain all 82 samples. Infant sex was included as an additional fixed effect together with age group, delivery mode, feeding type, maternal pre-pregnancy BMI, gestational weight gain, maternal GDM, maternal age, and parity, with infant identifier included as a random effect. This table shows only age-associated taxa, defined as features associated with the 6-month group at the exploratory threshold of $q < 0.25$. Direction refers to relative abundance at 6 months compared with 1 month. Infant sex was tested across 72 microbial features; no taxon was associated with infant sex after FDR correction at the exploratory threshold of $q < 0.25$ (minimum $q = 0.491$). Coefficients are shown to three decimal places; q values below 0.001 are shown as < 0.001.

Table S7. Sequencing depth and quality metrics across all shotgun metagenomic samples ($n = 82$).

Metric	Minimum	Maximum	Mean	Median
Raw reads	19,645,504	29,318,150	22,485,888	22,102,515
Raw bases (Gb)	5.93	8.85	6.79	6.67
Q30 (%)	93.15	96.32	94.53	94.59
Insert size (bp)	455	527	487.6	488
Read length (bp)	150	150	150	150

Note: Metrics are summarized across all 82 fecal metagenomic samples. No sample was excluded based on sequencing depth after library-level quality control.

Table S8. Catalog of metagenome-assembled genomes (MAGs) with CheckM2 quality metrics and GTDB-Tk taxonomy ($n = 171$).

MAG ID	Phylum	Genus	Compl. (%)	Contam. (%)	N50 (bp)
C1_4945_bin.13_sub	Actinomycetota	Actinomyces	55.8	0.22	5367
C1_787888_maxbin.003	Actinomycetota	Actinomyces	100.0	0.06	99201
C1_895275_maxbin.008	Actinomycetota	Aveggerthella	89.2	1.29	12927
C1_515077_bin.12	Actinomycetota	Bifidobacterium	100.0	0.08	320002
C1_537591_maxbin.002	Actinomycetota	Bifidobacterium	99.9	0.23	183545
C1_481334_bin.11	Actinomycetota	Bifidobacterium	100.0	0.28	242119
C1_4945_bin.9	Actinomycetota	Bifidobacterium	100.0	0.11	262073
C1_518880_maxbin.005	Actinomycetota	Bifidobacterium	99.9	0.87	154004
C1_171306_maxbin.005_sub	Actinomycetota	Bifidobacterium	96.6	7.85	29306
C1_369425_bin.15	Actinomycetota	Bifidobacterium	99.7	0.05	90030
C1_455613_maxbin.001	Actinomycetota	Bifidobacterium	100.0	0.66	309063
C1_667767_maxbin.004	Actinomycetota	Collinsella	94.9	2.06	229762
C1_471714_bin.1	Actinomycetota	Corynebacterium	100.0	0.10	297485
C1_930915_maxbin.001	Actinomycetota	Cutibacterium	100.0	0.19	388026
C1_787888_maxbin.002	Actinomycetota	Dermabacter	100.0	0.47	273807
C6_313477_bin.16	Actinomycetota	Eggerthella	65.8	0.06	237586
C6_930915_bin.10	Actinomycetota	Eggerthella	100.0	0.17	165174
C6_667767_bin.18_sub	Actinomycetota	Eggerthella	73.8	0.03	7073
C1_274915_bin.27	Actinomycetota	Enorma	71.8	0.16	326586
C6_313477_bin.13	Actinomycetota	Gleimia	99.9	0.05	262371
C1_518880_maxbin.002	Actinomycetota	Lawsonella	100.0	0.57	333903
C6_288604_maxbin.012_sub	Actinomycetota	Pauljensenia	75.2	2.02	5591
C1_286379_bin.11	Actinomycetota	Pauljensenia	99.8	0.28	243803
C1_902491_bin.14	Actinomycetota	Pauljensenia	100.0	0.88	68662
C6_194264_maxbin.007	Actinomycetota	Pauljensenia	100.0	0.08	40326
C6_376310_bin.5	Actinomycetota	Pauljensenia	74.2	0.46	6280
C6_385648_bin.14	Actinomycetota	Pauljensenia	99.9	1.21	24863
C6_930915_bin.6	Actinomycetota	Pauljensenia	99.8	0.22	38047
C1_99487_bin.13	Actinomycetota	Pauljensenia	84.7	1.78	12095
C6_385648_maxbin.008_sub	Actinomycetota	Rothia	80.6	3.37	14460
C1_99487_bin.7	Actinomycetota	Rothia	89.7	0.06	133575
C1_667767_maxbin.001	Actinomycetota	Rothia	100.0	0.01	614214
C1_314333_maxbin.004	Actinomycetota	Rothia	100.0	0.71	225413
C1_471714_maxbin.003	Actinomycetota	Scardovia	96.3	4.37	133641
C6_591898_bin.17	Actinomycetota	Senegalimassilia	77.7	1.73	6697
C1_930915_maxbin.002	Actinomycetota	Varibaculum	98.9	0.90	278320
C1_376310_bin.14	Bacillota	Alkalicoccus	99.7	0.22	70689
C6_733239_maxbin.010_sub	Bacillota	Alkalicoccus	47.5	2.95	1757
C6_286379_bin.10	Bacillota	Anaerostipes	100.0	0.74	264535
C6_455613_maxbin.001	Bacillota	Blautia	99.8	0.31	294401
C6_930915_bin.3	Bacillota	Blautia	100.0	4.27	176976
C6_313477_bin.10	Bacillota	Blautia	100.0	0.21	158660
C6_456528_bin.11	Bacillota	Blautia	66.4	1.15	209054
C6_337382_bin.4	Bacillota	Blautia	100.0	0.32	129069
C1_390713_maxbin.009_sub	Bacillota	Blautia	96.2	4.30	56269
C6_518880_bin.18	Bacillota	Blautia_A	46.4	0.26	68503
C6_271021_maxbin.003	Bacillota	Blautia_A	87.8	2.59	162439
C6_787888_bin.25	Bacillota	Blautia_A	41.5	0.00	55665
C6_306474_bin.8	Bacillota	Clostridioides	98.1	0.61	24388
C6_288604_bin.13	Bacillota	Clostridium	99.8	2.15	107315
C1_787888_bin.19	Bacillota	Clostridium	96.4	1.19	121348

MAG ID	Phylum	Genus	Compl. (%)	Contam. (%)	N50 (bp)
C6_375972_bin.6	Bacillota	Clostridium	100.0	1.96	57293
C1_667767_maxbin.025_sub	Bacillota	Clostridium	96.5	6.09	9712
C6_456528_bin.16	Bacillota	Clostridium	97.4	0.94	72688
C6_667767_bin.32	Bacillota	Clostridium_AP	91.2	0.02	16924
C6_930915_bin.8	Bacillota	Eisenbergiella	97.3	5.18	232394
C6_667767_bin.14	Bacillota	Eisenbergiella	99.1	11.18	230758
C1_518880_maxbin.001	Bacillota	Enterocloster	100.0	7.41	548606
C1_286974_bin.11	Bacillota	Enterococcus	100.0	0.10	329331
C6_930915_bin.5	Bacillota	Enterococcus_A	95.0	1.06	61240
C6_667767_bin.27	Bacillota	Enterococcus_A	100.0	1.83	127979
C1_639490_bin.11_sub	Bacillota	Enterococcus_A	99.1	5.59	33390
C1_895275_bin.13	Bacillota	Enterococcus_B	100.0	0.80	49277
C1_518880_bin.6	Bacillota	Enterococcus_B	100.0	0.39	142141
C6_733239_bin.9	Bacillota	Enterococcus_B	100.0	0.09	77138
C6_313477_bin.20	Bacillota	Enterococcus_C	99.9	0.00	326172
C6_313477_bin.22	Bacillota	Enterococcus_D	100.0	0.31	378661
C6_271021_bin.12	Bacillota	Enterococcus_D	99.8	0.45	33832
C6_286379_bin.9	Bacillota	Eubacterium	84.4	0.47	363148
C6_518880_bin.17	Bacillota	Faecalimonas	100.0	0.88	109178
C6_455613_bin.24	Bacillota	Faecalimonas	90.6	0.00	144929
C1_283204_bin.24	Bacillota	Finegoldia	82.4	0.28	9632
C1_99487_bin.17	Bacillota	Finegoldia	86.0	0.65	65658
C1_274915_bin.28	Bacillota	Flavonifractor	99.0	0.75	89602
C6_306035_maxbin.001	Bacillota	Fusicatenibacter	100.0	0.56	221129
C6_902491_maxbin.003	Bacillota	Gemmiger	82.5	2.66	56816
C6_456528_maxbin.014_sub	Bacillota	Hominilimicola	52.2	1.28	2263
C6_306474_bin.4	Bacillota	Hungatella	99.9	8.22	156734
C6_787888_maxbin.014_sub	Bacillota	Intestinibacter	88.2	0.48	35847
C6_902491_bin.18	Bacillota	Intestinibacter	95.7	0.95	9459
C1_283204_bin.26	Bacillota	Lacticaseibacillus	99.1	0.07	35979
C6_591898_bin.7	Bacillota	Lacticaseibacillus	100.0	0.03	85357
C1_375741_bin.17	Bacillota	Lactobacillus	100.0	0.00	298721
C6_71516_bin.3	Bacillota	Lactobacillus	100.0	0.15	440951
C1_667767_bin.23	Bacillota	Lactococcus	100.0	0.21	81827
C6_902491_bin.6	Bacillota	Leuconostoc	81.2	1.41	9915
C6_71516_bin.24_sub	Bacillota	Ligilactobacillus	99.9	0.01	89176
C6_71516_maxbin.015	Bacillota	Limosilactobacillus	99.4	0.51	38370
C1_99487_maxbin.002	Bacillota	Limosilactobacillus	94.2	0.08	190718
C1_471714_bin.6	Bacillota	Limosilactobacillus	63.0	0.16	54889
C1_306474_bin.8	Bacillota	Limosilactobacillus	71.0	2.16	5542
C6_271021_bin.4_sub	Bacillota	Mediterraneibacter	80.0	0.02	117718
C1_537591_maxbin.005	Bacillota	Mediterraneibacter	99.9	1.33	220130
C6_390713_maxbin.011_sub	Bacillota	Mediterraneibacter	71.2	8.96	83628
C6_337382_maxbin.010_sub	Bacillota	Megasphaera	81.4	7.76	72684
C6_71516_bin.8	Bacillota	Megasphaera	96.7	0.01	99575
C6_390713_bin.20	Bacillota	Merdimonas	91.5	0.00	178398
C1_639490_maxbin.010	Bacillota	Negativicoccus	82.6	0.00	34785
C6_456528_bin.12	Bacillota	Otoolea	95.4	0.00	151742
C1_286379_bin.17_sub	Bacillota	Otoolea	86.1	1.97	55240
C6_375972_bin.10	Bacillota	Peptoniphilus_A	97.9	0.18	30097
C1_194264_maxbin.011_sub	Bacillota	Phascolarctobacterium	78.3	4.28	4891
C6_667767_bin.13	Bacillota	Robinsoniella	96.8	12.38	27722

MAG ID	Phylum	Genus	Compl. (%)	Contam. (%)	N50 (bp)
C6_787888_bin.33	Bacillota	Roseburia	91.1	0.00	134081
C1_566463_bin.2	Bacillota	Ruthenibacterium	97.6	0.40	40619
C6_366577_bin.17	Bacillota	SIG603	96.5	3.10	49333
C1_787888_bin.12	Bacillota	Sarcina	97.0	0.44	1874830
C6_366577_maxbin.018_sub	Bacillota	Sarcina	72.1	6.02	2615
C6_337382_bin.19	Bacillota	Scatavimonas	100.0	0.82	90781
C6_306474_maxbin.002_sub	Bacillota	Sellimonas	48.6	1.43	153078
C1_733239_maxbin.008_sub	Bacillota	Staphylococcus	54.9	7.52	2118
C1_314333_bin.2	Bacillota	Staphylococcus	95.7	0.20	305649
C1_667767_bin.27	Bacillota	Staphylococcus	100.0	1.91	115566
C1_930915_maxbin.010_sub	Bacillota	Staphylococcus	58.5	7.18	4007
C1_314333_bin.5	Bacillota	Staphylococcus	100.0	0.09	126058
C1_446448_bin.16	Bacillota	Staphylococcus	100.0	0.01	129232
C1_515077_bin.16	Bacillota	Staphylococcus	93.0	1.65	14842
C1_288604_bin.17	Bacillota	Staphylococcus	81.4	0.21	97552
C1_409111_bin.10	Bacillota	Streptococcus	92.2	1.64	36057
C1_171306_bin.4	Bacillota	Streptococcus	84.0	0.49	11141
C1_375741_bin.7_sub	Bacillota	Streptococcus	95.4	3.41	19300
C1_787888_bin.8	Bacillota	Streptococcus	99.9	0.01	73770
C6_591898_bin.10	Bacillota	Streptococcus	99.9	1.05	82459
C1_288604_bin.21	Bacillota	Streptococcus	100.0	0.10	94785
C6_518880_bin.4	Bacillota	Streptococcus	100.0	0.61	410256
C6_366577_maxbin.017_sub	Bacillota	Streptococcus	80.9	4.86	4692
C1_274915_bin.4	Bacillota	Streptococcus	100.0	0.01	84797
C1_518880_bin.11	Bacillota	Streptococcus	74.6	0.20	111987
C1_566463_bin.4	Bacillota	Streptococcus	89.7	0.52	13438
C6_667767_bin.38	Bacillota	Veillonella	100.0	0.32	30583
C1_515077_bin.2_sub	Bacillota	Veillonella	72.3	0.06	116647
C1_455613_bin.6	Bacillota	Veillonella	99.6	0.89	79203
C1_471714_bin.8	Bacillota	Veillonella	99.9	0.08	34831
C1_286379_bin.2_sub	Bacillota	Veillonella	66.9	0.28	69074
C6_194264_maxbin.004_sub	Bacillota	Veillonella_A	88.8	5.38	130735
C6_71516_maxbin.011_sub	Bacillota_I	Clostridium_AQ	99.0	3.17	100757
C1_286379_bin.10	Bacillota_I	Coprobacillus	97.3	1.53	76980
C6_787888_bin.21	Bacillota_I	Thomasclavelia	100.0	4.16	169461
C6_667767_bin.16	Bacillota_I	Thomasclavelia	88.7	0.42	31399
C6_384723_bin.4	Bacteroidota	Alistipes	99.9	0.28	216099
C1_455613_bin.2	Bacteroidota	Bacteroides	99.9	0.12	258133
C1_313477_bin.3	Bacteroidota	Bacteroides	100.0	0.39	222560
C1_515077_bin.8	Bacteroidota	Bacteroides	99.9	2.54	291433
C1_418147_bin.10	Bacteroidota	Bacteroides	92.1	0.66	171737
C6_71516_bin.5	Bacteroidota	Bacteroides	98.1	0.25	166667
C1_337382_bin.16	Bacteroidota	Bacteroides	77.5	0.22	83105
C1_418147_bin.4	Bacteroidota	Bacteroides	94.9	0.38	67499
C6_455613_bin.13	Bacteroidota	Bacteroides	96.3	0.38	154791
C1_194264_maxbin.005_sub	Bacteroidota	Bacteroides	77.7	9.09	166873
C1_566463_bin.10	Bacteroidota	Dysgonomonas_A	96.8	0.86	29173
C1_337382_bin.21	Bacteroidota	Parabacteroides	96.3	0.99	151794
C1_274915_bin.12_sub	Bacteroidota	Parabacteroides	93.7	0.28	23673
C1_274915_bin.16	Bacteroidota	Phocaeicola	85.5	0.09	51675
C1_667767_bin.16	Bacteroidota	Phocaeicola	100.0	0.06	145287
C1_902491_maxbin.002	Pseudomonadota	Enterobacter	98.2	4.15	133422

MAG ID	Phylum	Genus	Compl. (%)	Contam. (%)	N50 (bp)
C1_733239_bin.9	Pseudomonadota	Escherichia	100.0	0.00	427525
C6_375741_maxbin.010_sub	Pseudomonadota	Escherichia	78.7	5.87	2490
C1_283204_bin.10	Pseudomonadota	Haemophilus_D	99.9	0.02	42276
C6_385648_bin.3	Pseudomonadota	Haemophilus_D	84.9	0.16	20370
C1_787888_bin.4	Pseudomonadota	Haemophilus_D	100.0	1.04	58697
C1_4945_maxbin.002_sub	Pseudomonadota	Haemophilus_D	85.4	1.62	217991
C6_902491_bin.32	Pseudomonadota	Haemophilus_D	98.2	1.09	14600
C6_787888_bin.20	Pseudomonadota	Haemophilus_D	98.0	0.19	23877
C1_348475_bin.11	Pseudomonadota	Haemophilus_D	99.6	0.68	205479
C6_390713_maxbin.017	Pseudomonadota	Haemophilus_D	80.4	2.13	3918
C1_171306_bin.6	Pseudomonadota	Klebsiella	95.2	1.15	13361
C1_271021_bin.11	Pseudomonadota	Klebsiella	99.3	0.04	214959
C1_114694_maxbin.001	Pseudomonadota	Klebsiella	100.0	0.32	226808
C1_376310_maxbin.003	Pseudomonadota	Serratia	93.8	0.11	189815
C6_286379_maxbin.004	Verrucomicrobiota	Akkermansia	100.0	0.13	189145
C6_384723_maxbin.006	Verrucomicrobiota	Akkermansia	100.0	0.11	175970

Table S9. Genome-resolved host attribution of β-lactamase genes detected by RGI/CARD on metagenome-assembled genomes (MAGs).

β-lactamase gene	CARD AMR gene family	Host MAG (GTDB taxonomy)	Identity (%)	RGI cut-off
CblA-1	CblA β-lactamase	Bacteroides uniformis	99.3	Strict
CepA-44	CepA β-lactamase	Bacteroides fragilis	99.7	Strict
CfiA4	CfiA β-lactamase	Bacteroides hominis	100.0	Strict
CfxA3	CfxA β-lactamase	Bacteroides thetaiotaomicron	100.0	Strict
DYB-1	DYB β-lactamase	Dysgonomonas_A capnocytophagoides	98.8	Strict
ACT-45	ACT β-lactamase	Enterobacter hormaechei_C	100.0	Strict
CMY-189	CMY β-lactamase	Escherichia coli	76.1	Strict
EC-8	EC β-lactamase	Escherichia coli	98.1	Strict
CMY-190	CMY β-lactamase	Klebsiella aerogenes	78.7	Strict
LEN-16	LEN β-lactamase	Klebsiella variicola	100.0	Strict
SHV-71	SHV β-lactamase	Klebsiella pneumoniae	100.0	Strict
CTX-M-14	CTX-M β-lactamase	Sarcina perfringens[a]	100.0	Strict
SRT-3	SRT β-lactamase	Serratia ureilytica	100.0	Strict
PC1	BlaZ β-lactamase	Staphylococcus aureus	97.2	Strict
PC1 beta-lactamase (blaZ)	BlaZ β-lactamase	Staphylococcus epidermidis	94.7	Strict

Note: Only hydrolytic β-lactamase genes are listed. Penicillin-binding protein (PBP3) target mutations (n = 14) and porin/efflux determinants conferring reduced β-lactam susceptibility are not hydrolytic β-lactamases and are excluded. No β-lactamase gene was detected in any of the eight Bifidobacterium MAGs. Identity denotes percentage identity to the best-hit CARD reference.

[a] The single CTX-M-14 hit occurred on an unplaced contig within a Firmicutes (Clostridiaceae) bin, most consistent with misbinning of a mobile element rather than genuine carriage by this taxon.

Table S10. Mobile genetic context of the β-lactamase-carrying contigs (geNomad plasmid/provirus classification).

β-lactamase gene	Host MAG (genus)	geNomad class	Plasmid score	Plasmid contig (kb)
CblA-1	Bacteroides	Chromosomal[a]	–	–
CepA-44	Bacteroides	Chromosomal[a]	–	–
CfiA4	Bacteroides	Chromosomal[a]	–	–
CfxA3	Bacteroides	Chromosomal[a]	–	–
DYB-1	Dysgonomonas_A	Chromosomal[a]	–	–
ACT-45	Enterobacter	Chromosomal[a]	–	–
CMY-189	Escherichia	Chromosomal[a]	–	–
EC-8	Escherichia	Chromosomal[a]	–	–
CMY-190	Klebsiella	Plasmid	0.988	30.3
LEN-16	Klebsiella	Plasmid[c]	0.986	477.3
SHV-71	Klebsiella	Plasmid	0.986	146.7
CTX-M-14	Sarcina	Unplaced[b]	–	–
SRT-3	Serratia	Plasmid	0.934	89.8
PC1	Staphylococcus	Chromosomal[a]	–	–
blaZ (PC1)	Staphylococcus	Chromosomal[a]	–	–

Note: Assembled contigs were classified with geNomad (v1.12.0; end-to-end mode), which identified 930 plasmid and 675 proviral (prophage) contigs across the 171 MAGs. Of the 15 hydrolytic β-lactamase genes (Table S9), 4 were located on plasmid-classified contigs, all within Enterobacteriaceae MAGs. No β-lactamase gene was located on a predicted proviral contig. Conjugation hallmark genes were not detected on the four bla-bearing plasmid contigs. "–" indicates not applicable.

[a] Contig not classified as a plasmid or provirus by geNomad, consistent with a chromosomal context.

[b] The single CTX-M-14 hit occurred on the unplaced Firmicutes-bin contig noted in Table S9 (most consistent with misbinning of a mobile element) and was not classified as a plasmid.

[c] The LEN-16-carrying contig (477 kb, non-circular, 1 plasmid hallmark) is atypically large for a plasmid; given that LEN-type enzymes are intrinsic chromosomal β-lactamases of Klebsiella variicola, this plasmid classification is uncertain and may reflect a chromosomal fragment.

2 SUPPLEMENTARY FIGURES

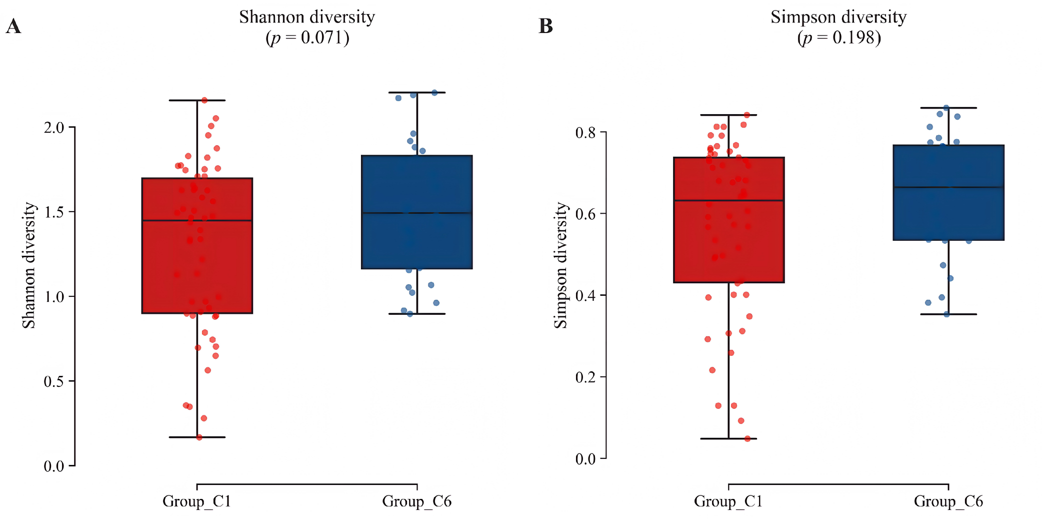

Figure S1. Comparison of alpha diversity indices (Shannon and Simpson) between C1 and C6 groups. Comparison of (A) Shannon index and (B) Simpson index between C1 and C6 groups. Unlike species richness, both Shannon (p = 0.071) and Simpson (p = 0.198) indices showed no significant differences between groups, indicating stable community evenness despite the increase in species number.

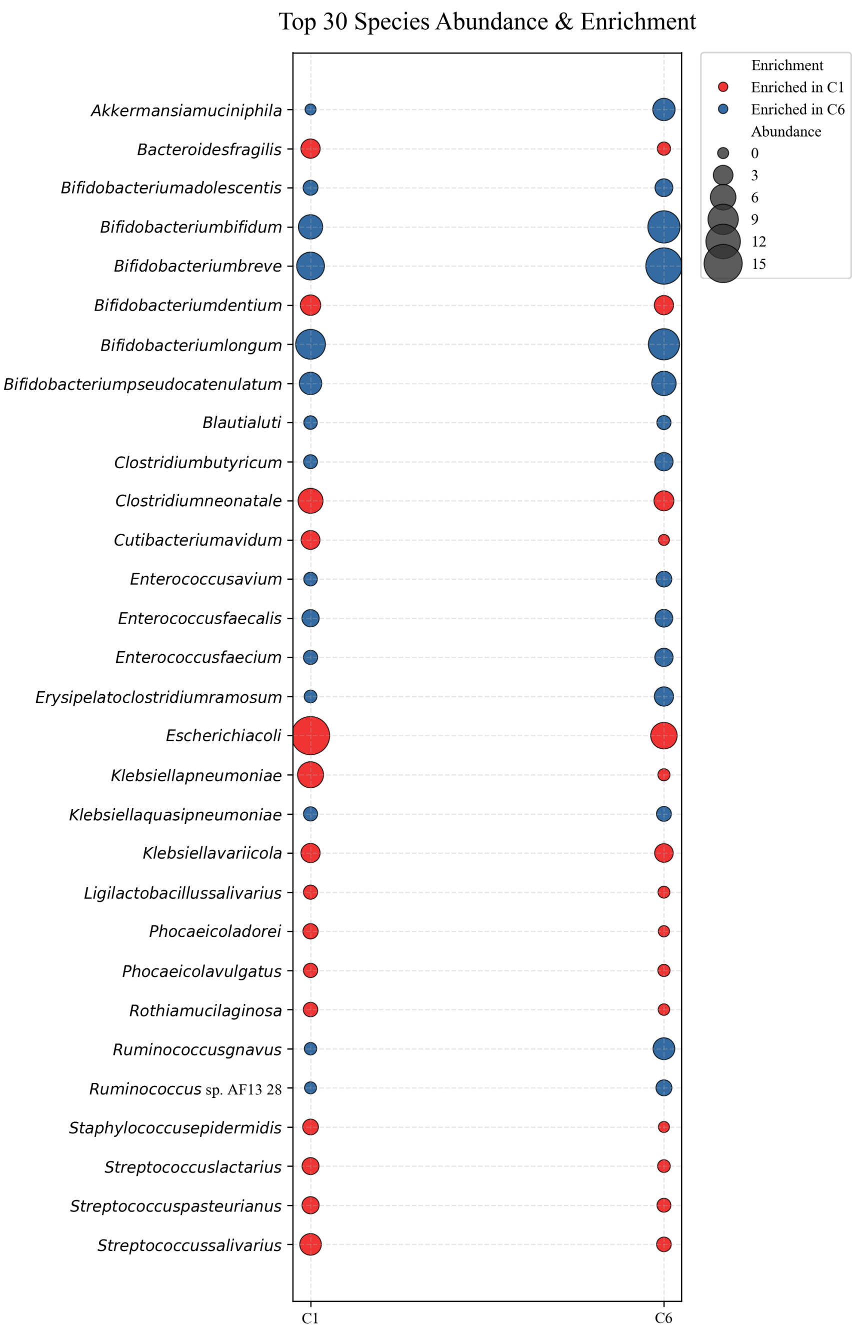

Figure S2. Bubble plot of core infant gut microbiota. Visualization of the top 30 most abundant bacterial species. Bubble size represents relative abundance; color indicates overall enrichment direction (Red: C1; Blue: C6).

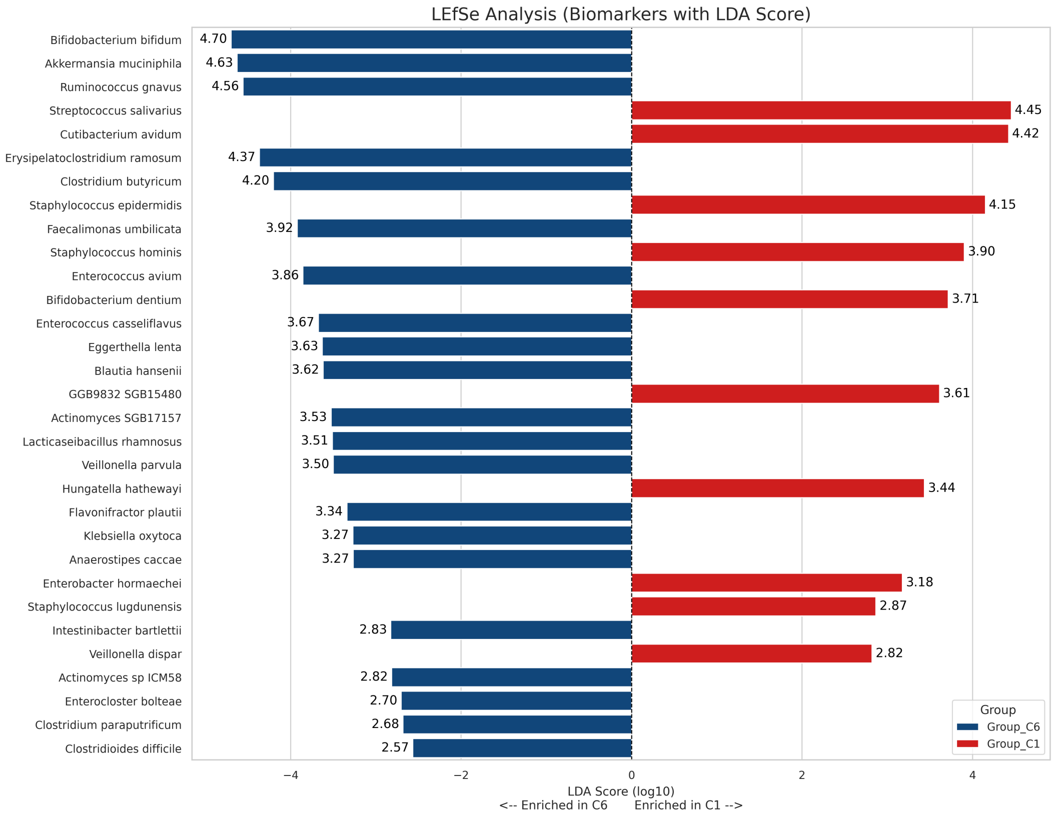

Figure S3. Differentially abundant taxa between C1 and C6 groups identified by LEfSe. Linear discriminant analysis effect size (LEfSe) identifying differentially abundant taxa between age groups (LDA score > 2.0). Blue bars indicate species enriched in the C6 group; red bars indicate species enriched in the C1 group.

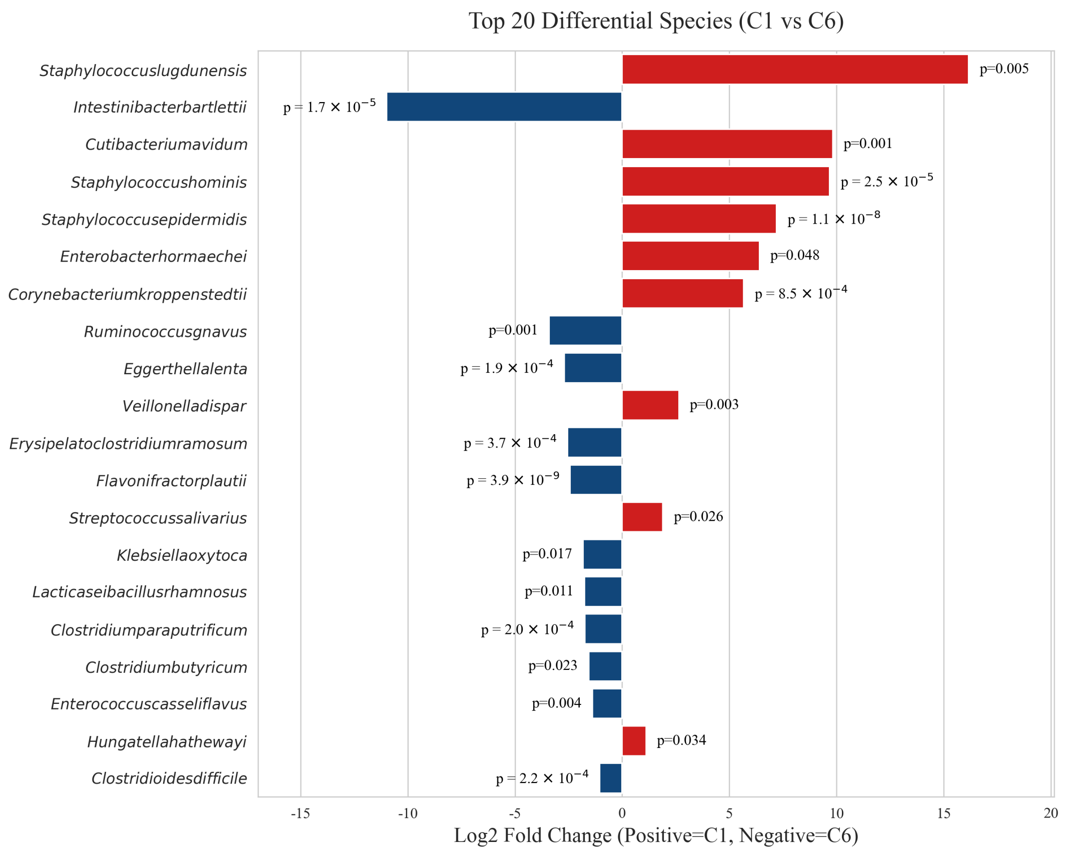

Figure S4. Detailed profiles of age-discriminatory taxa. Bar plot showing the top 20 bacterial species with the most significant abundance differences between C1 and C6, ranked by absolute effect size (Log_2 Fold Change). Species enriched in C1 and C6 are indicated in red (positive values) and blue (negative values), respectively.

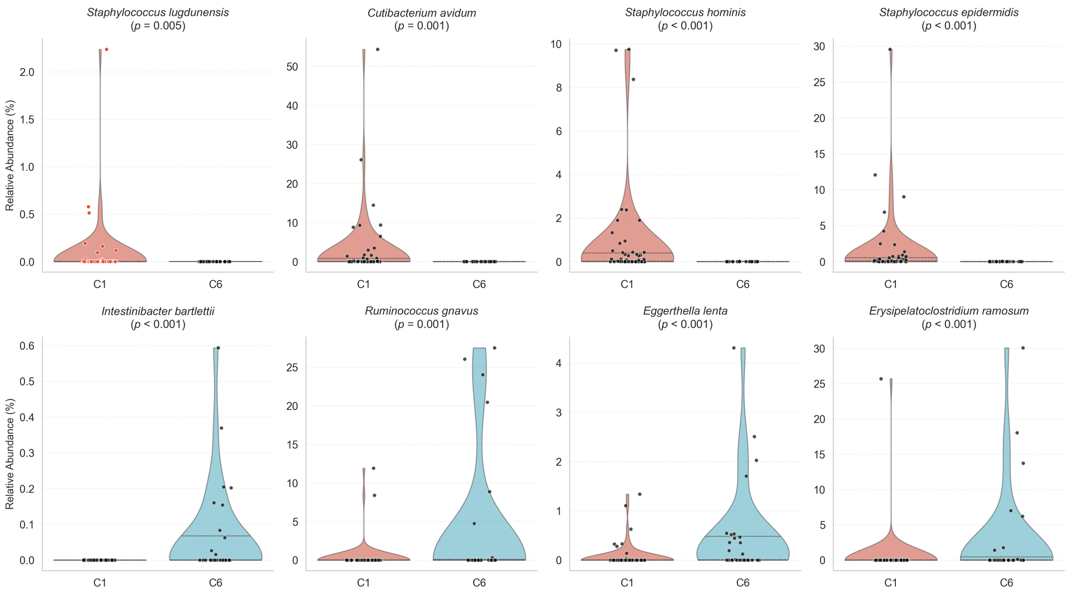

Figure S5. Violin plots of key age-discriminatory taxa. Detailed abundance distributions of 8 representative biomarkers identified by MaAsLin2 and LEfSe. (Top row) Species enriched in C1 (e.g., Staphylococcus epidermidis, Streptococcus salivarius) showing depletion over time. (Bottom row) Species enriched in C6 (e.g., Granulicatella, Bifidobacterium bifidum) showing colonization. Violin shapes represent data density; points represent individual samples.

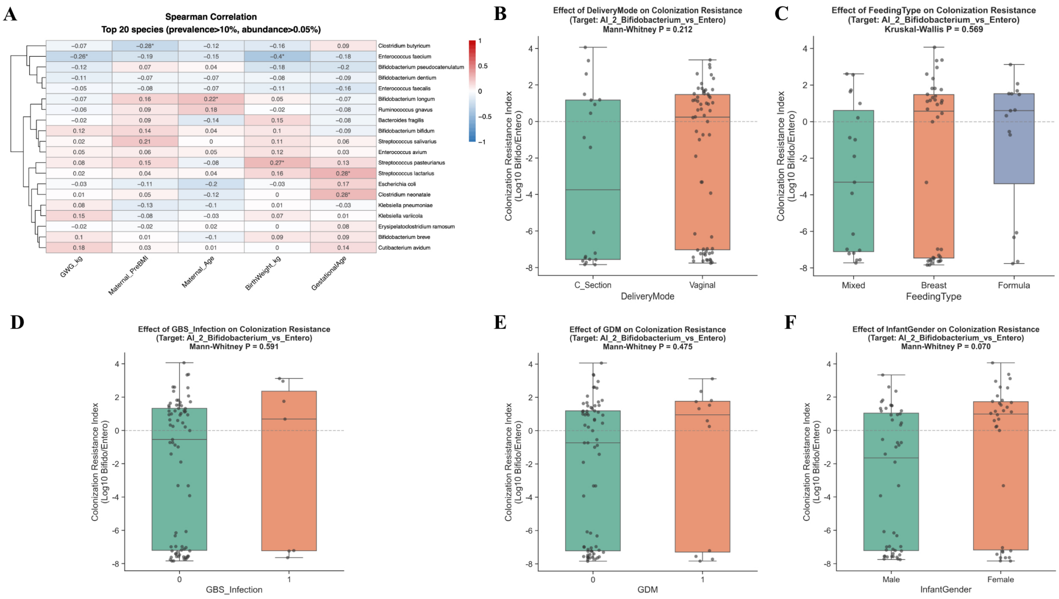

Figure S6. Impact of perinatal clinical factors on colonization-resistance proxy indices and species-level microbiota composition. (A) Spearman correlation heatmap between the relative abundances of MaAsLin2-significant species and continuous perinatal clinical variables, including gestational weight gain (GWG), maternal pre-pregnancy BMI, maternal age, birth weight, and gestational age. (B–F) Boxplots showing the Colonization Resistance Index (AI) stratified by (B) delivery mode, (C) feeding type, (D) maternal GBS status, (E) maternal GDM status, and (F) infant sex. No statistically significant differences in AI scores were observed across these clinical strata (all $p > 0.05$). These stratified analyses were descriptive and should not be interpreted as excluding possible effects of perinatal or clinical factors. Asterisks denote statistically significant correlations ($* \ p < 0.05$).

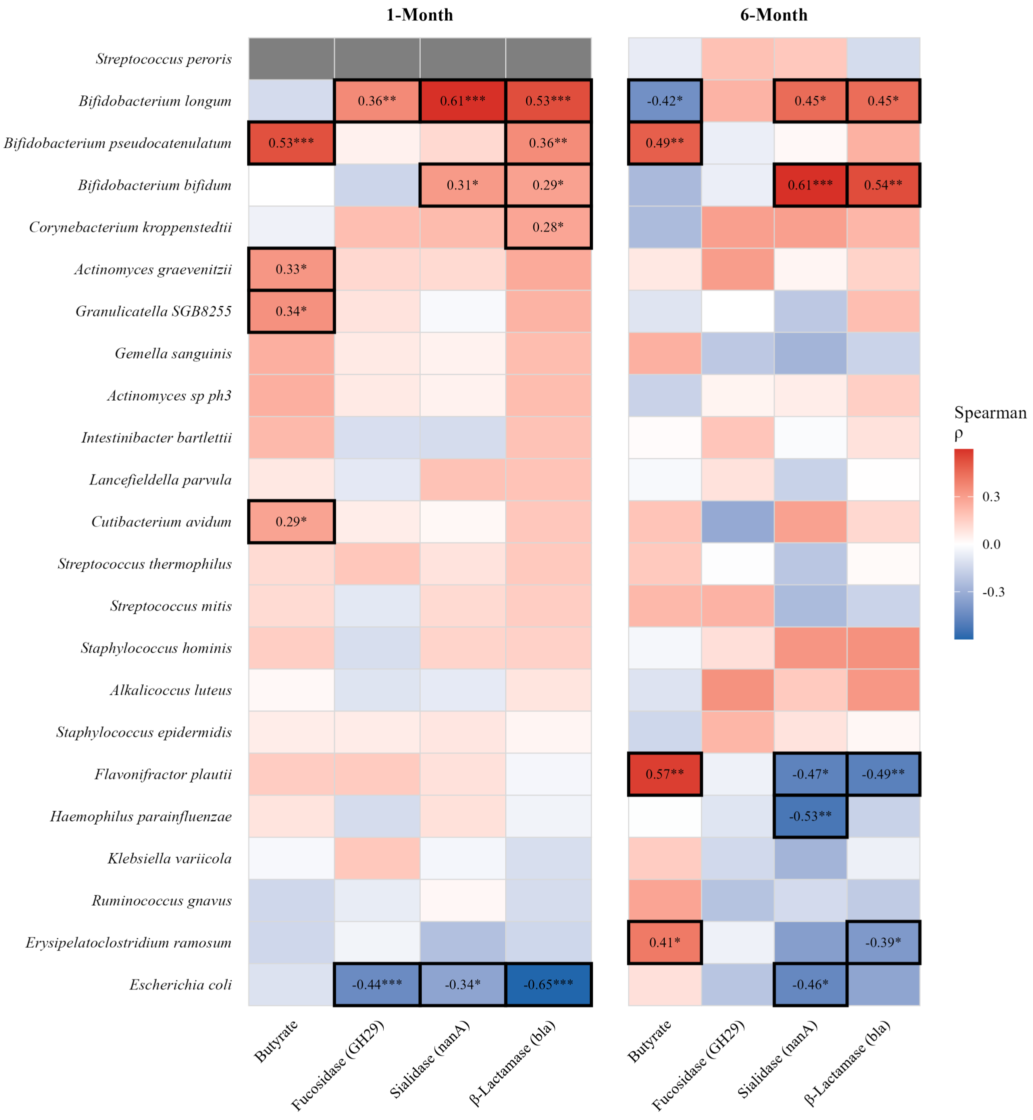

Figure S7. Exploratory Spearman correlation analysis between key bacterial species and functional markers. Spearman correlation coefficients were calculated separately for infants at 1 month (C1, $n = 54$) and 6 months (C6, $n = 28$). The heatmap shows correlations between selected bacterial species and functional markers, including butyrate biosynthesis, HMO-related enzymes including fucosidase GH29 and sialidase nanA, and CPM-normalized β-lactamase (bla) relative abundance. The color gradient represents Spearman's correlation coefficient (ρ), with red indicating positive correlations and blue indicating negative correlations. Cells with statistically significant correlations are outlined with bold black borders; asterisks denote significance levels (* $p < 0.05$, ** $p < 0.01$, *** $p < 0.001$).

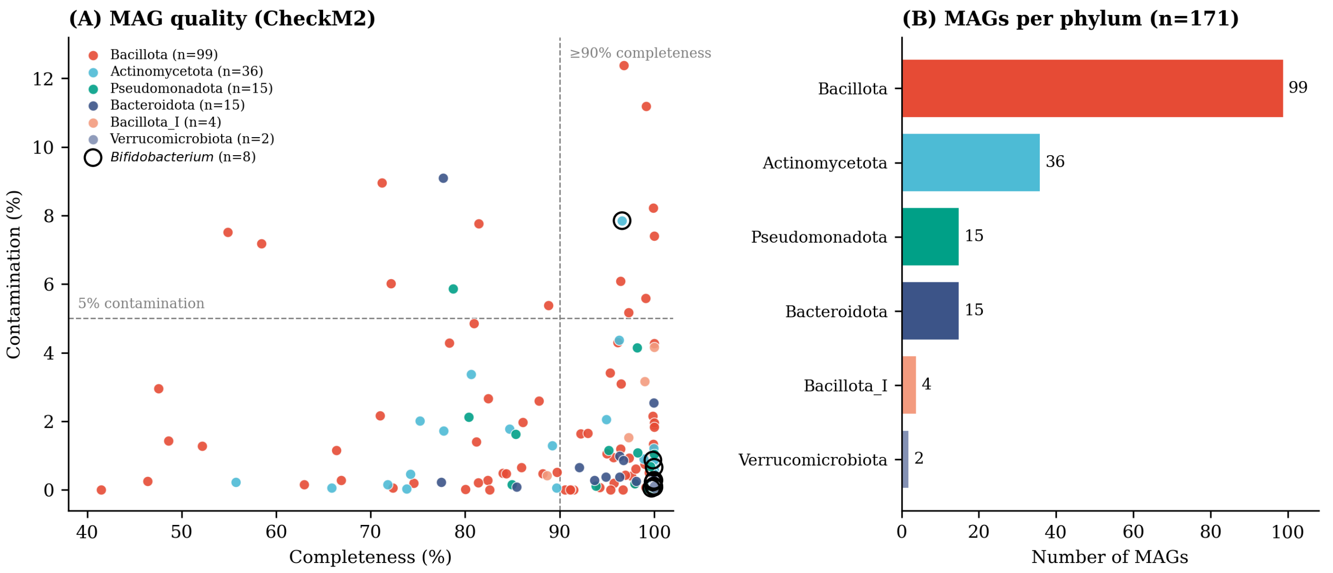

Figure S8. Quality and phylum-level composition of the 171 recovered MAGs. (A) CheckM2 completeness versus contamination for all 171 MAGs, colored by phylum; Bifidobacterium MAGs are circled. Dashed lines mark the ≥90% completeness and ≤5% contamination thresholds used to define near-complete genomes. (B) Number of MAGs recovered per phylum.

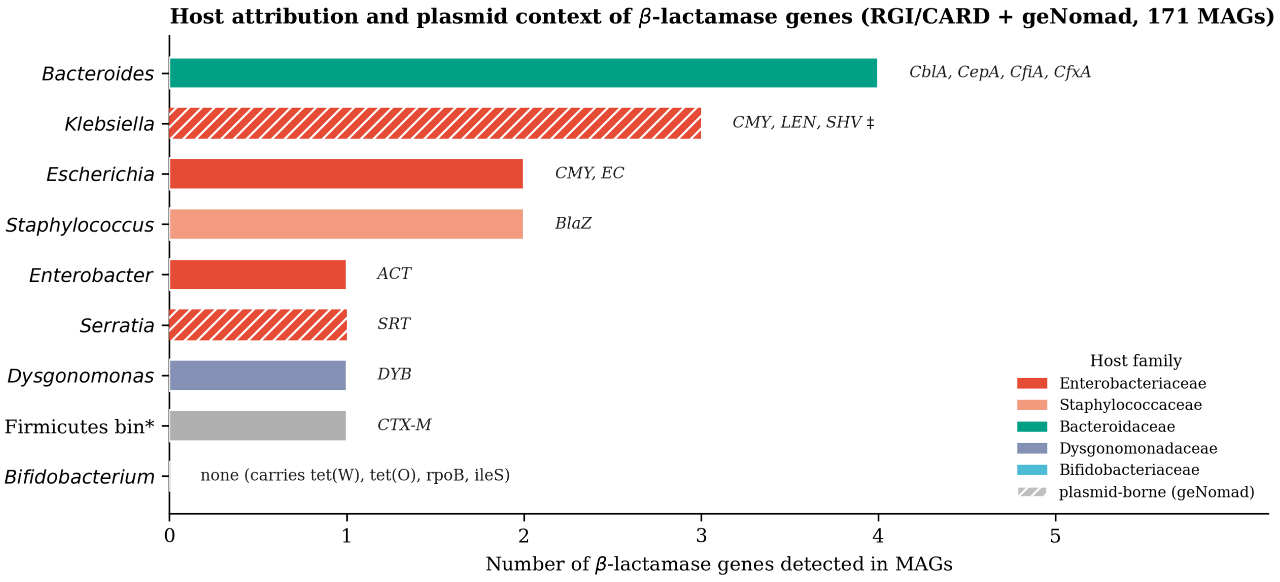

Hatched = β-lactamase gene on a geNomad plasmid-classified contig; ‡ LEN-16 on an atypically large (477 kb) contig, plasmid status uncertain.

**single CTX-M-14 hit on an unplaced contig in a Firmicutes bin, most consistent with misbinning of a mobile element*

Figure S9. Host attribution and plasmid context of β-lactamase genes. Number of hydrolytic β-lactamase genes detected by RGI/CARD per host genus across the 171 MAGs, colored by host family and annotated with the corresponding CARD gene families. Hatched bar segments denote genes located on geNomad plasmid-classified contigs; all four plasmid-borne genes were within Enterobacteriaceae (Klebsiella and Serratia), and no β-lactamase gene was located on a predicted proviral contig. The LEN-16-carrying contig (‡) was atypically large (477 kb) and its plasmid classification is uncertain. No β-lactamase gene was detected in any of the eight Bifidobacterium MAGs (which carried tet(W), tet(O), rpoB, and ileS instead). The single CTX-M-14 hit occurred on an unplaced contig within a Firmicutes bin (asterisk), most consistent with misbinning of a mobile element. Penicillin-binding protein target mutations and porin/efflux determinants are not hydrolytic β-lactamases and are excluded. Per-gene mobile genetic context is provided in Table S10.
